# Associations between gut microbiota and gynecological cancers: A bi-directional two-sample Mendelian randomization study

**DOI:** 10.1097/MD.0000000000037628

**Published:** 2024-03-29

**Authors:** Youqian Kong, Shaoxuan Liu, Xiaoyu Wang, Rui Qie

**Affiliations:** a Graduate School, Heilongjiang University of Chinese Medicine, Harbin, China; b Department of Internal Medicine, First Affiliated Hospital, Heilongjiang University of Chinese Medicine, Harbin, China.

**Keywords:** causality, gut microbiota, gynecological cancer, Mendelian randomization, SNPs

## Abstract

Growing evidence has suggested that gut microbiota is associated with gynecologic cancers. However, whether there is a causal relationship between these associations remains to be determined. A two-sample Mendelian randomization (MR) evaluation was carried out to investigate the mechanism associating gut microbiota and 3 prevalent gynecological cancers, ovarian cancer (OC), endometrial cancer, and cervical cancer as well as their subtypes in individuals of European ancestry. The Genome-wide association studies statistics, which are publically accessible, were used. Eligible instrumental single nucleotide polymorphisms that were significantly related to the gut microbiota were selected. Multiple MR analysis approaches were carried out, including inverse variance weighted, MR-Egger, Weighted Median methods, and a range of sensitivity analyses. Lastly, we undertook a reverse MR analysis to evaluate the potential of reverse causality. We sifted through 196 bacterial taxa and identified 33 suggestive causal relationships between genetic liability in the gut microbiota and gynecological cancers. We found that 11 of these genera could be pathogenic risk factors for gynecological cancers, while 19 could lessen the risk of cancer. In the other direction, gynecological cancers altered gut microbiota composition. Our MR analysis revealed that the gut microbiota was causally associated with OC, endometrial cancer, and cervical cancer. This may assist in providing new insights for further mechanistic and clinical studies of microbiota-mediated gynecological cancer.

## 1. Introduction

Gynecological cancers, encompassing ovarian cancer (OC), cervical cancer (CC), and endometrial cancer (EC), etc present a formidable health crisis globally. According to GLOBOCAN 2020 cancer incidence and mortality estimates,^[1]^ out of the approximately 19.3 million fresh cancer diagnoses and close to 10 million new cancer fatalities globally in 2020, these 3 cancer types account for nearly 7% of new cases and deaths.^[1]^ OC ranks among the most prevalent cancers afflicting women worldwide and has the greatest mortality rate among all reproductive system cancers.^[2]^ CC, despite being one of the most preventable cancers, consistently emerges as the second leading cause of cancer-related deaths in women aged 20 to 39 years.^[3]^ Further, the escalating global obesity epidemic coupled with an aging population has triggered a stark rise in the incidence of EC.^[4]^ Unmistakably, gynecological cancers constitute a grave threat to women’s health. Research efforts to identify specific biomarkers and devise effective therapeutic strategies for prompt diagnosis and accurate therapeutic monitoring in the disease’s early stages continue unabated, motivated by the need to enhance patient survival and the still unclear pathogenesis and criteria for early diagnosis.

The gut microbiota is regarded as a dynamic and multifaceted entity.^[5]^ Over 22 million genes have been identified within the gut microbiome and it has further been found that certain microbial subgroups can exert a direct influence on human physiology via their metabolites.^[6]^ Maintaining gut microbiota equilibrium is crucial for human health. Dysbiosis of the gut flora can precipitate a range of deleterious effects, such as instigating imbalances in inflammatory and immune responses, DNA damage, increased intestinal permeability, and abnormal estrogen levels, thereby fostering carcinogenesis.^[7]^ This carcinogenic influence has been extensively explored in preclinical and clinical studies especially gastrointestinal and respiratory tract tumors.^[8–11]^ Changes in gut and vaginal microbiome composition have been associated with virtually all gynecological cancers (e.g., ovarian, uterine, cervical, vaginal, and vulvar cancers, etc).^[12,13]^ A burgeoning body of research suggests that individual microbiota differences might correlate with varying disease susceptibility.^[14]^ The gut microbiome might not only serve as a diagnostic marker but also as a potential therapeutic target.^[15]^ However, due to objective factors such as sample collection or processing, data handling, and technical and research methods, as well as sample size limitations and the complex interrelationships between various bacterial genera, the majority of current studies are confined to animal experiments and small clinical cohort trials,^[15,16]^ yielding somewhat limited results.

Mendelian randomization (MR) is a method that employs genetic variation associated with specific exposure factors to investigate the causal effect of modifiable exposure factors (i.e., potential risk factors) on disease.^[17]^ MR enables a more robust causality study than traditional observational studies and mitigates bias arising from confounding factors.^[18]^ Over the past decade, MR has been successfully utilized across various fields, including cardiovascular diseases,^[19]^ metabolic disorders,^[20]^ and cancer,^[21]^ to uncover causal relationships and inform clinical decision-making.

This study harnesses a two-sample MR analysis to probe the causal relationship between specific microbial taxa and the risk of OC, EC, and CC using pooled statistics from a large genome-wide association study (GWAS), identify specific genera within the gut flora that might wield a causative influence on these 3 gynecological malignancies, and assess the potential impact of the 3 cancers on the gut microbiota by reverse MR.

## 2. Methods

### 2.1. Study design

**Figure 1.**
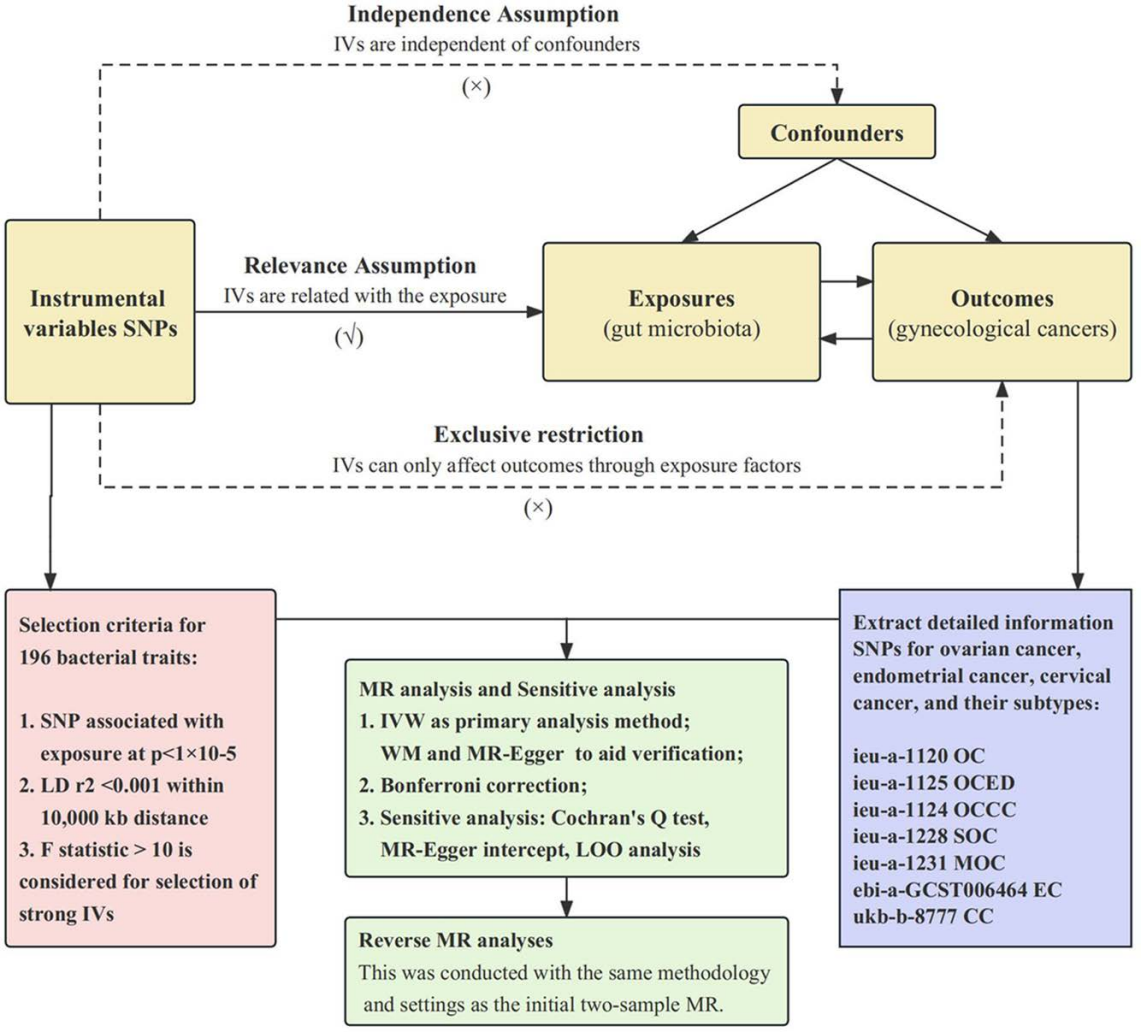
The flowchart of the study: the whole workflow of MR analysis. GWAS = genome-wide association studies; CC = cervical cancer; EC = endometrial cancer; IVs = instrumental variables; IVW = inverse variance weighted; LOO = leave-one-out; MR = Mendelian randomization; OC = ovarian cancer; SNPs = single nucleotide polymorphisms.

Employing a two-sample MR framework, we investigated the genetic correlation between gut microbiota and gynecological cancers. Our focus was to determine if gut microbiota exerts a causal influence on 3 prevalent gynecological cancers: OC, EC, and CC, including their respective subtypes. Complementarily, a reverse MR analysis was conducted to examine the potential influence of gynecological cancers on the composition of pathogenic gut microbiota. To assure the validity of our MR analysis, we adhered to 3 pivotal assumptions: (i) There exists a robust, statistically significant association between the exposure variable and the instrumental variables (IVs), (ii) Both the exposure variable and any potential confounders of the outcome are independent of the IVs, exerting no influence on them, (iii) The exposure variable acts as the exclusive mediator in the association between the IVs and the outcome, with no other variables intervening in this relationship. Our study design is delineated graphically in Figure 1.

In conducting our analysis, we utilized publicly available GWAS summary statistics. Given that our study was predicated exclusively on these preexisting, anonymized data sets, there was no requirement for the collection of fresh data or the procurement of additional ethical clearance.

### 2.2. Data sources

#### 2.2.1. Gut microbiota.

Data pertaining to genetic variations in human gut microbiota were sourced from the summary statistics of the MiBioGen study,^[22]^ the largest multi-ethnic meta-analysis of gut microbiota to date. The study involved 18,340 participants from diverse origins including Europeans, US Hispanics/Latinos, East Asians, and others. These participants were recruited across 24 cohorts in the MiBioGen consortium, spanning multiple countries: the USA, Canada, Israel, South Korea, Germany, Denmark, the Netherlands, Belgium, Sweden, Finland, and the UK. The study encompassed 211 bacterial taxa, including 131 genera, 35 families, 20 orders, 16 classes, and 9 phyla. The analysis, however, concentrated on 196 bacterial traits after excluding 15 traits with unidentified species (unknown family or genus). To analyze the microbial composition, 3 variable regions of the 16S rRNA gene (V1–V2, V3–V4, and V4) were utilized.^[23]^ The GWAS data can be accessed at https://mibiogen.gcc.rug.nl/.

#### 2.2.2. Gynecological cancers

Data for OC genetics were acquired from the epithelial ovarian cancer GWAS study,^[24]^ facilitated by the Ovarian Cancer Association Consortium. The study encompassed 66,450 European-derived samples, with 25,509 cases of OC. A subset of 20,791 women diagnosed with the invasive disease from 7 genotyping studies was evaluated against 40,941 control subjects of European lineage. It is important to note that only samples with more than 80% European ancestry were included in the analyses. For EC risk estimates, we consulted a large-scale GWAS consisting of the Endometrial Cancer Association Consortium, the UK Biobank, and the Epidemiology of Endometrial Cancer Consortium,^[25]^ comprising 12,906 EC cases and 108,979 controls, all of the European ancestry. Histological subtyping of EC cases resulted in 8758 endometrioid and 1230 non-endometrioid cases. The contributing cohorts for this study spanned multiple countries, including Australia, Belgium, Germany, Sweden, the UK, and the USA. Finally, aggregate GWAS statistics for CC were acquired from the Medical Research Council-Integrative Epidemiology Unit Open GWAS database, specifically the UK Biobank dataset, which is a vast prospective cohort study, includes over half a million UK participants and amasses extensive data on lifestyle, risk factors, and health outcomes.^[26]^ This dataset contains 1889 CC cases and 461,044 controls. The GWAS data for these cancers are stored in the IEU Open GWAS project (https://gwas.mrcieu.ac.uk/). Detailed information about the GWAS samples used in this study is provided in Table 1.

**Table 1:** Gynecological cancers genome-wide association study samples used in this study.

GWAS ID	Trait	No. case	No. control	Sample size	Year	Consortium	Populations	Reference
ieu-a-1120	OC	25,509	40,941	66,450	2017	OCAC	European	Phelan et al^[24]^
ieu-a-1125	OCED	2810	40,941	43,751				
ieu-a-1124	OCCC	1366	40,941	42,307				
ieu-a-1228	SOC	14,049	40,941	54,990				
ieu-a-1122	LGSOC	1012	40,941	41,953				
ieu-a-1121	HGSOC	13,037	40,941	53,978				
ieu-a-1231	MOC	2566	40,941	43,507				
ieu-a-1232	LMPMOC	1149	40,941	42,090				
ieu-a-1123	IMOC	1417	40,941	42,358				
ebi-a-GCST006464	EC	12,906	108,979	121,885	2018	NA	European	O’Mara et al^[25]^
ebi-a-GCST006465	ECEH	8758	46,126	54,884				
ebi-a-GCST006466	ECNEH	1230	35,447	36,677				
ukb-b-8777	CC	1889	461,044	462,933	2018	MRC-IEU	European	UKBB (data filed: 20001)

CC = cervical cancer, EC = endometrial cancer, ECEH = endometrial cancer endometrioid histology, ECNEH = endometrial cancer non-endometrioid histology, GWAS = genome-wide association study, HGSOC = high grade serous ovarian cancer, IMOC = invasive mucinous ovarian cancer, LGSOC = low grade serous ovarian cancer, LMPMOC = ow malignant potential mucinous ovarian cancer, MRC-IEU = Medical Research Council Integrative Epidemiology Unit; OC = ovarian cancer, OCAC = Ovarian Cancer Association Consortium, OCCC = clear cell ovarian cancer, OCED = endometrioid ovarian cancer, SOC = serous ovarian cancer, UKBB = UK Biobank.

### 2.3. Selection of genetic instrumental variables

We implemented stringent quality control measures to select genetic predictors associated with microbiome characteristics, thereby ensuring accuracy in the elucidation of a causal relationship between gut microbiota and gynecological cancers. Initially, we established genome-wide significance thresholds of *P* < 5 × 10^-8^ to identify single nucleotide polymorphisms (SNPs) closely associated with gut microbiota and gynecologic cancers. To obtain more comprehensive results, we used a relatively relaxed threshold of *P* < 1 × 10^-5^, given the limited number of eligible IVs.^[27]^ We performed a linkage disequilibrium analysis based on the European Thousand Genomes Project to meet the assumption of MR and excluded SNPs that did not meet the criteria (R^2^ < 0.001, clumping distance = 10,000 kb) from the analysis. Additionally, SNPs with minor allele frequencies <0.01 were excluded from the analysis. When SNPs associated with exposure variables were missing from the resulting GWAS, we selected alternative SNPs with high linkage disequilibrium (r^2^ > 0.80) to ensure full coverage.

Moreover, we calculated the F statistic using the equation $F=R^{2}\times \left(N-2\right)/\left(1-R^{2}\right)$ to assess instrumental strength, where R^2^ = proportion of variance. N = sample size. A value of <10 for the F statistic indicates a higher likelihood of weak instrumental bias.^[28]^

### 2.4. Statistical analysis

#### 2.4.1. MR analysis.

A thorough investigation of the potential causal associations between gut microbiota and prevalent gynecological cancers was conducted utilizing 5 renowned MR methods: inverse variance weighted (IVW),^[29]^ maximum likelihood,^[30]^ MR-Egger,^[31]^ weighted median,^[32]^ and weighted mode.^[33]^ Our approach was tailored to maximize the robustness of our results and minimize false positives. The prime method employed was the IVW, chosen for its capacity to provide more conservative and reliable estimates under certain conditions compared to other methods.^[34]^ The remaining methods were employed for cross-validation to ascertain the consistency of the findings. We considered the nominal significance level for MR estimates of *P* < .05. If *P* < .05 for the IVW method and the other methods had an effect in the same direction, it was considered that there could be a potential causal relationship between gut microbiota and the outcome. Results were considered more robust if *P* < .05 for 2 or more MR methods.^[35]^

Multiple-testing significance was determined at each taxonomic level using Bonferroni correction,^[27,36]^ with thresholds set at 0.05 divided by the effective number of independent bacterial taxa: phylum (*P* = .05/9 = 5.56 × 10^−3^), class (*P* = .05/16 = 3.13 × 10^−3^), order (*P* = .05/20 = 2.50 × 10^−3^), family (*P* = .05/35 = 1.43 × 10^−3^), and genus (*P* = .05/131 = 3.82 × 10^−4^). For the primary MR results, *P*-values below the Bonferroni-corrected *P*-value were considered significant causal associations, and *P*-values below .05 but above the corrected were considered suggestive causal associations.^[37]^ The relationship between human gut microbiota and the risk of gynecological cancers was represented as odds ratio (OR) with their corresponding 95% confidence intervals (CI).

#### 2.4.2. Sensitivity analysis

For both significant and nominal significant causalities, we assessed the heterogeneity of the IVW and MR-Egger estimates using the Cochran Q test.^[38]^ MR-Egger regression was designed to assess potential pleiotropy bias.^[31]^ In addition, to assess the stability of the results, we performed a “leave-one-out” analysis by excluding one SNP at a time to explore if a single SNP dominated the inference of causal associations.^[39]^

#### 2.4.3. Reverse MR analysis

To explore whether gynecological cancers have any potential causal effects on the gut microbiota, we performed a reverse MR analysis (i.e., each gynecological cancer as the exposure, and the gut microbiota as the outcome) using SNPs that are associated with cancers as IVs. This reverse MR analysis was conducted with the same methodology and settings as the initial two-sample MR.

All statistical analyses were performed using the Two-Sample MR package in R statistical software (version 4.3.0).

## 3. Results

### 3.1. Causal effects of gut microbiota on gynecological cancers

In total, 196 bacterial taxa (9 phyla, 16 classes, 20 orders, 32 families, and 119 genera) were included for MR analysis. Following a meticulous selection process, 895 SNPs were chosen as instrumental variables. The number of SNPs associated with each bacterial taxon varied, ranging from 2 to 17 (Table S1, Supplemental Digital Content, http://links.lww.com/MD/M3). Significantly, all F-statistics surpassed 10, suggesting the absence of weak instrumental variables in our study.

### 3.2. Ovarian cancer

We found that the genetically predicted *genus Victivallis* was associated with a higher risk of OC (OR = 1.10, 95% CI = 1.00–1.20, *P* = .039 > 3.82 × 10^−4^) in the IVW method, the association was also stable in the weighted median method and the maximum likelihood method. Three taxon of gut microbes were suggestively associated with endometrioid ovarian cancer using IVW method: *family Streptococcaceae* (OR = 1.64, 95% CI = 1.13–2.39, *P* = .009 > 1.43 × 10^−3^), *genus Adlercreutzia* (OR = 1.53, 95% CI = 1.15–2.04, *P* = .004), and *genus Ruminiclostridium6* (OR = 1.36, 95% CI = 1.07–1.72, *P* = .012); In contrast, the *genus Oscillibacter* was negatively associated with clear cell ovarian cancer risk (OR = 0.70, 95% CI = 0.52–0.95, *P* = .021).

In the serous ovarian cancer (SOC) and its subtype analysis, 3 gut microbial abundances were associated with a low risk of them: *family Alcaligenaceae* with SOC (OR = 0.82, 95% CI = 0.69–0.98, *P* = .025), *genus Corinthobacter* with low grade serous ovarian cancer (LGSOC) (OR = 0.52, 95% CI = 0.29–0.93, *P* = .029), *family Alcaligenaceae* with high grade serous ovarian cancer (HGSOC) (OR = 0.81, 95% CI = 0.68–0.97, *P* = .019). On the other hand, 4 gut microbial abundances were associated with high risk: *genus Romboutsia* with SOC (OR = 1.17, 95% CI = 1.01–1.34, *P* = .031), *genus Peptococcu*s (OR = 1.40, 95% CI = 1.02–1.91, *P* = .036) and *phylum Cyanobacteria* (OR = 1.56, 95% CI = 1.03–2.36, *P* = .036 > 5.56 × 10^−3^) with LGSOC, *genus Romboutsia* with HGSOC (OR = 1.15, 95% CI = 1.00–1.33, *P* = .049). Among them, we found that the *P*-value of *family Alcaligenaceae* for both SOC and HGSOC was <0.05, with the OR values and confidence intervals being close to each other, as verified by the IVW, Maximum likelihood and weighted median methods, and the causal relationships were all consistent with each other as a potential protective factor. Similarly, *genus Romboutsia* produced similar estimates for SOC and HGSOC in the IVW and weighted median methods, and genus Romboutsia may be one of the important potential risk factors.

**Figure 2.**
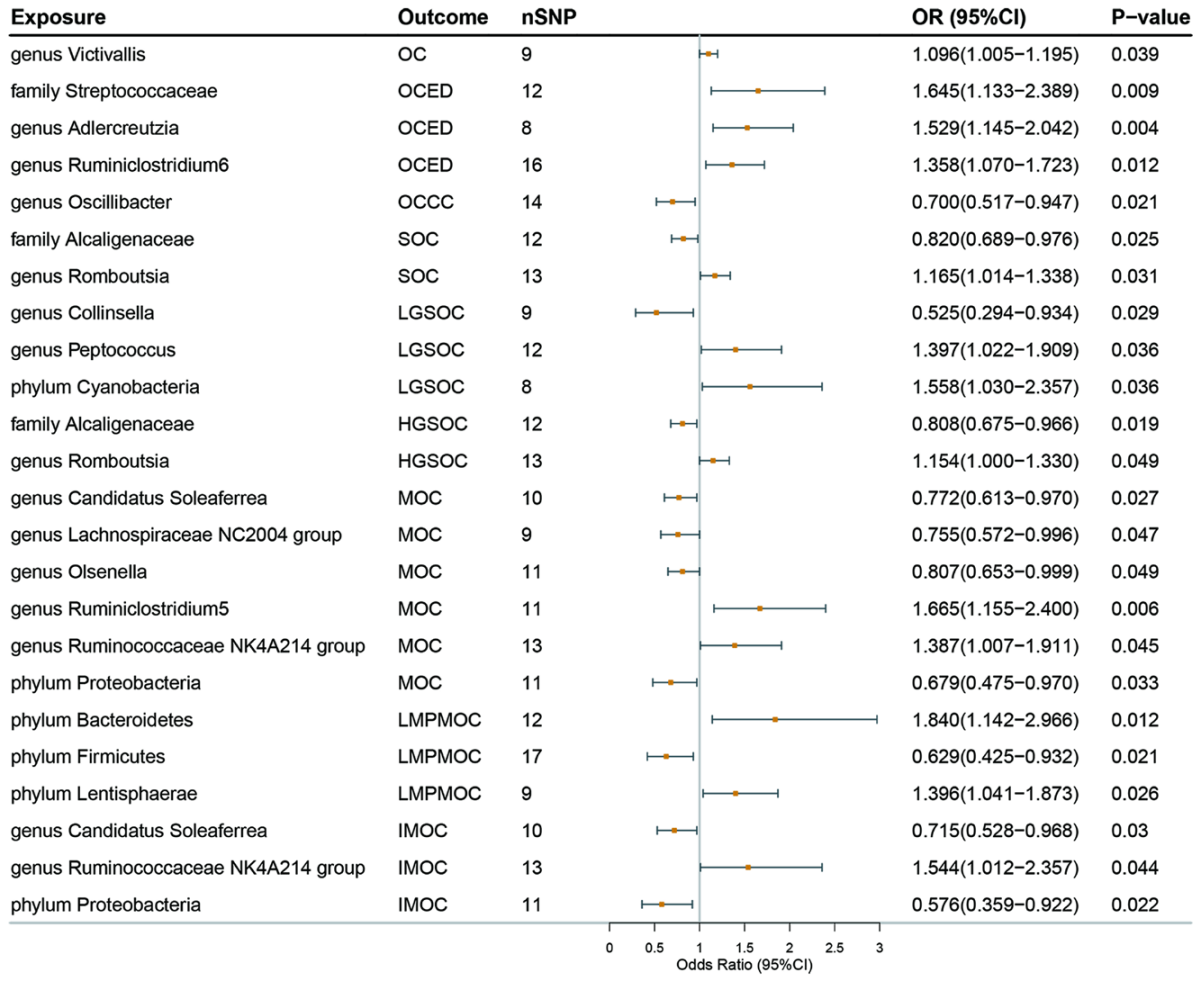
Forest plot of the association between gut microbiome and OC and its subtypes based on the IVW MR model. CI = confidence interval; HGSOC = high grade serous ovarian cancer; IMOC = invasive mucinous ovarian cancer; LGSOC = low grade serous ovarian cancer; LMPMOC = low malignant potential mucinous ovarian cancer; OC = ovarian cancer; OCED = endometrioid ovarian cancer; OCCC = clear cell ovarian cancer; OR = odds ratio; SNPs = Single nucleotide polymorphisms; SOC = serous ovarian cancer.

In addition, in the analysis of mucinous ovarian cancer (MOC), 4 gut microbial abundances were associated with a low risk of them: *genera Candidatus* (OR = 0.77, 95% CI = 0.61–0.97, *P* = .027), *genera Lachnospiraceae NC2004 group* (OR = 0.76, 95% CI = 0.57–1.00, *P* = .047), *genera Olsenella* (OR = 0.81, 95% CI = 0.65–1.00, *P* = .049), *phylum Proteobacteria* (OR = 0.68, 95% CI = 0.48–0.97, *P* = .033). Furthermore, 2 gut microbial abundances were associated with a high risk: *genus Ruminococcus 5* (OR = 1.67, 95% CI = 1.16–2.40, *P* = .006), *genus RuminococcaceaeNK4A214 group* (OR = 1.39, 95% CI = 1.01–1.91, *P* = .045). We found that the gut microbial potentially causally related to Invasive mucinous ovarian cancer (IMOC) was the same as MOC and that the IVW analysis estimates were close, 3 with IMOC: *genus Candidatus Soleaferrea* (OR = 0.71, 95% CI = 0.53–0.97, *P* = .030), *genus Ruminococcus in the group NK4A214* (OR = 1. 54, 95% CI = 1.01–2.36, *P* = .044), and *phylum Proteobacteria* (OR = 0.58, 95% CI = 0.36–0.92, *P* = .022) were associated. In contrast, 3 taxa of gut microbes were associated with low malignant potential mucinous ovarian cancer are different from MOC: *genus Candidatus Soleaferrea* (OR = 0.71, 95% CI = 0.53–0.97, *P* = .030), *genus Ruminococcus* in the group NK4A214 (OR = 1. 54, 95% CI = 1.01–2.36, *P* = .044), and *phylum Proteobacteria* (OR = 0.58, 95% CI = 0.36–0.92, *P* = .022). However, we did not find a significant causal relationship between gut microbiota and ovarian cancer after Bonferroni testing, and the results as suggestive causal associations. Table 2 and Figure 2 present the potential causal relationships between specific gut microbes and OC, as well as its subtypes. Refer to Table S2, Supplemental Digital Content, http://links.lww.com/MD/M4 for further details.

**Table 2:** Mendelian randomization estimates for the association between gut microbiota and ovarian cancer.

Bacterial taxa (exposure)	Outcome	MR method	No. SNP	*P*-value	OR	95%CI
Genus Victivallis	OC	IVW	9	.039	1.10	1.00–1.20
Family Streptococcaceae	OCED	IVW	12	.009	1.64	1.13–2.39
Genus Adlercreutzia	OCED	IVW	8	.004	1.53	1.15–2.04
Genus Ruminiclostridium6	OCED	IVW	16	.012	1.36	1.07–1.72
Genus Oscillibacter	OCCC	IVW	14	.021	0.70	0.52–0.95
Family Alcaligenaceae	SOC	IVW	12	.025	0.82	0.69–0.98
Genus Romboutsia	SOC	IVW	13	.031	1.17	1.01–1.34
Genus Collinsella	LGSOC	IVW	9	.029	0.52	0.29–0.93
Genus Peptococcus	LGSOC	IVW	12	.036	1.40	1.02–1.91
Phylum Cyanobacteria	LGSOC	IVW	8	.036	1.56	1.03–2.36
Family Alcaligenaceae	HGSOC	IVW	12	.019	0.81	0.68–0.97
Genus Romboutsia	HGSOC	IVW	13	.05	1.15	1.00–1.33
Genus Candidatus Soleaferrea	MOC	IVW	10	.027	0.77	0.61–0.97
Genus Lachnospiraceae NC2004 group	MOC	IVW	9	.047	0.76	0.57–1.00
Genus Olsenella	MOC	IVW	11	.049	0.81	0.65–1.00
Genus Ruminiclostridium5	MOC	IVW	11	.006	1.67	1.16–2.40
Genus Ruminococcaceae NK4A214 group	MOC	IVW	13	.045	1.39	1.01–1.91
Phylum Proteobacteria	MOC	IVW	11	.033	0.68	0.48–0.97
Phylum Bacteroidetes	LMPMOC	IVW	12	.012	1.84	1.14–2.97
Phylum Firmicutes	LMPMOC	IVW	17	.021	0.63	0.42–0.93
Phylum Lentisphaerae	LMPMOC	IVW	9	.026	1.40	1.04–1.87
Genus Candidatus Soleaferrea	IMOC	IVW	10	.03	0.71	0.53–0.97
Genus Ruminococcaceae NK4A214 group	IMOC	IVW	13	.044	1.54	1.01–2.36
Phylum Proteobacteria	IMOC	IVW	11	.022	0.58	0.36–0.92

CI = confidence interval, HGSOC = high grade serous ovarian cancer, IMOC = invasive mucinous ovarian cancer; IVW = inverse variance weighted, LGSOC = low grade serous ovarian cancer, LMPMOC = low malignant potential mucinous ovarian cancer, MR = Mendelian randomization, OC = ovarian cancer, OCCC = clear cell ovarian cancer, OCED = endometrioid ovarian cancer, OR = odds ratio, SNPs = single nucleotide polymorphisms, SOC = serous ovarian cancer.

### 3.3. Endometrial cancer

**Figure 3.**
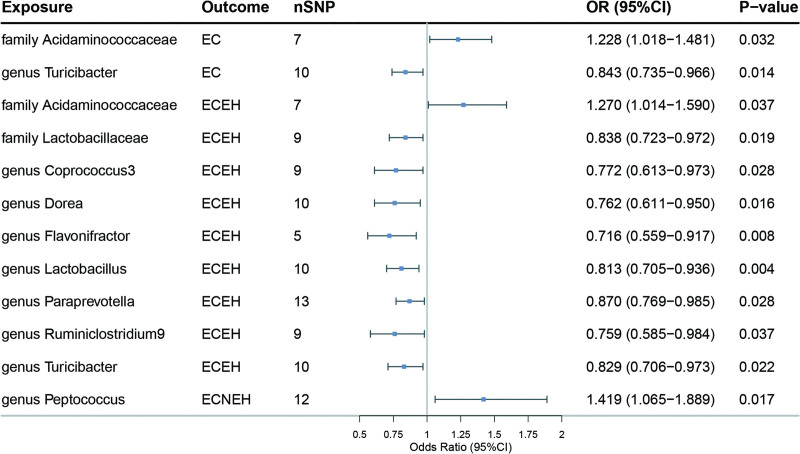
Forest plot of the association between gut microbiome and EC and its subtypes based on the IVW MR model. CI, confidence interval; ECEH, endometrial cancer endometrioid histology; ECNEH, endometrial cancer non-endometrioid histology; OR = odds ratio; SNPs = single nucleotide polymorphisms.

Meanwhile, the identical approaches were utilized to explore the causal effect of gut microbiome on EC and its subtypes, 12 suggestive causal relationships were found after Bonferroni correction. We found that 2 taxa of gut microbes in the IVW method: *family Acidaminococcaceae* (OR = 1.23, 95% CI = 1.02–1.48, *P* = .032), and *genus Turicibacter* (OR = 0.84, 95% CI = 0.73–0.97, *P* = .014) to be suggestively associated with EC. There were 8 gut microbial abundances were potentially associated with a low risk of endometrial cancer endometrioid histology (ECEH) and can be considered suggestive protective factors: *family Lactobacillaceae* (OR = 0.84, 95% CI = 0.72–0.97, *P* = .019), *genus Coprococcus3* (OR = 0.77, 95% CI = 0.61–0.97, *P* = .028), *genus Dorea* (OR = 0.76, 95% CI = 0.61–0.95, *P* = .016), *genus Flavonifractor* (OR = 0.72, 95% CI = 0.56–0.92, *P* = .008), *genus Lactobacillus* (OR = 0.81, 95% CI = 0.71–0.94, *P* = .004), *genus Paraprevotella* (OR = 0.87, 95% CI = 0.77–0.98, *P* = .028), *genus Ruminiclostridium9* (OR = 0.76, 95% CI = 0.59–0.98, *P* = .037), and *genus Turicibacter* (OR = 0.83, 95% CI = 0.71–0.97, *P* = .022). On the other hand, *family Acidaminococcaceae* (OR = 1.27, 95% CI = 1.01–1.59, *P* = .037) was potentially associated with a high risk of ECEH. As for endometrial cancer non-endometrioid histology, we found that one taxon of gut microbes was associated with: *genus Peptococcus* (OR = 1.42, 95% CI = 1.07–1.89, *P* = .017). In particular, *genus Flavonifractor* was negatively associated with ECEH risk in the IVW method and the results remained stable in the maximum likelihood and weighted median validation. The results are shown in Table 3 and Figure 3. Refer to Table S2, Supplemental Digital Content, http://links.lww.com/MD/M4 for further details.

**Table 3:** Mendelian randomization estimates for the association between gut microbiota and endometrial cancer.

Bacterial taxa (exposure)	Outcome	MR method	No. SNP	*P*-value	OR	95%CI
Family Acidaminococcaceae	EC	IVW	7	.032	1.228	1.02–1.48
Genus Turicibacter	EC	IVW	10	.014	0.843	0.73–0.97
Family Acidaminococcaceae	ECEH	IVW	7	.037	1.270	1.01–1.59
Family Lactobacillaceae	ECEH	IVW	9	.019	0.838	0.72–0.97
Genus Coprococcus3	ECEH	IVW	9	.028	0.772	0.61–0.97
Genus Dorea	ECEH	IVW	10	.016	0.762	0.61–0.95
Genus Flavonifractor	ECEH	IVW	5	.008	0.716	0.56–0.92
Genus Lactobacillus	ECEH	IVW	10	.004	0.813	0.71–0.94
Genus Paraprevotella	ECEH	IVW	13	.028	0.870	0.77–0.98
Genus Ruminiclostridium9	ECEH	IVW	9	.037	0.759	0.59–0.98
Genus Turicibacter	ECEH	IVW	10	.022	0.829	0.71–0.97
Genus Peptococcus	ECNEH	IVW	12	.017	1.419	1.07–1.89

CI = confidence interval, EC = endometrial cancer, ECEH = endometrial cancer endometrioid histology, ECNEH = endometrial cancer non-endometrioid histology, IVW = inverse variance weighted, MR = Mendelian randomization, OR = odds ratio, SNPs = single nucleotide polymorphisms.

### 3.4. Cervical cancer

**Figure 4.**
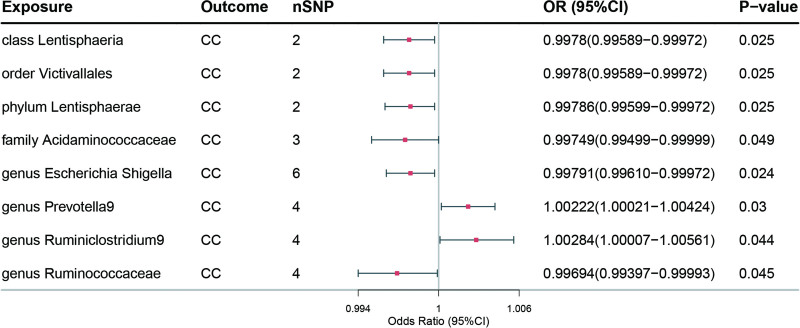
Forest plot of the association between gut microbiome and CC based on the IVW MR model. CC = cervical cancer; CI = confidence interval; OR = odds ratio; SNPs = single nucleotide polymorphisms.

Similarly, the analysis revealed suggestive causal associations between certain gut microbes and the risk of CC. In the IVW method, it was discovered that 6 gut microbes, namely *class Lentisphaeria* (OR = 0.9978, 95% CI = 0.9959–0.9997, *P* = .025 > 3.13 × 10^−3^), *order Victivallales* (OR = 0.9978, 95% CI = 0.9959–0.9997, *P* = .025 > 2.50 × 10^−3^), *phylum Lentisphaerae* (OR = 0.9979, 95% CI = 0.9960–0.9997, *P* = .025), *family Acidaminococcaceae* (OR = 0.9975, 95% CI = 0.9950–1.0000, *P* = .049), *genus Escherichia Shigella* (OR = 0.9979, 95% CI = 0.9961–0.9997, *P* = .024), and *genus Ruminococcaceae UCG005* (OR = 0.9969, 95% CI = 0.9940–0.9999, *P* = .045) were associated with a reduced risk of CC. In contrast, 2 gut microbes, including *genus Prevotella9* (OR = 1.0022, 95% CI = 1.0002–1.0042, *P* = .030), *genus Ruminiclostridium9* (OR = 1.0028, 95% CI = 1.0001–1.0056, *P* = .044) were associated with an increased risk of CC. The results are shown in Table 4 and Figure 4. Refer to Table S2, Supplemental Digital Content, http://links.lww.com/MD/M4 for further details.

**Table 4:** Mendelian randomization estimates for the association between gut microbiota and cervical cancer.

Bacterial taxa (exposure)	Outcome	MR method	No. SNP	*P*-value	OR	95%CI
Class Lentisphaeria	CC	IVW	2	.025	0.9978	0.9959–0.9997
Order Victivallales	CC	IVW	2	.025	0.9978	0.9959–0.9997
Phylum Lentisphaerae	CC	IVW	2	.025	0.9979	0.9960–0.9997
Family Acidaminococcaceae	CC	IVW	3	.049	0.9975	0.9950–1.0000
Genus Escherichia Shigella	CC	IVW	6	.024	0.9979	0.9961–0.9997
Genus Prevotella9	CC	IVW	4	.03	1.0022	1.0002–1.0042
Genus Ruminiclostridium9	CC	IVW	4	.044	1.0028	1.0001–1.0056
Genus Ruminococcaceae UCG005	CC	IVW	4	.045	0.9969	0.9940–0.9999

CC = cervical cancer, CI = confidence interval, IVW = inverse variance weighted, MR = Mendelian randomization, OR = odds ratio, SNPs = single nucleotide polymorphisms.

All MR methods produced consistent direction of effect estimates. We found the results of the Cochrane Q statistics showed no significant heterogeneity (*P* > .05). In addition, no evidence of horizontal pleiotropy for gut microbiota in gynecological cancers with *P* > .05 when using the MR-Egger regression intercept approach (Table S3, Supplemental Digital Content, http://links.lww.com/MD/M5). Additionally, the leave-one-out analysis further supported that none of the identified causal associations were driven by any single IV (Figure S1, Supplemental Digital Content, http://links.lww.com/MD/M9).

### 3.5. Bi-directional causal effects between gut microbiota and gynecological cancers risk

**Figure 5.**
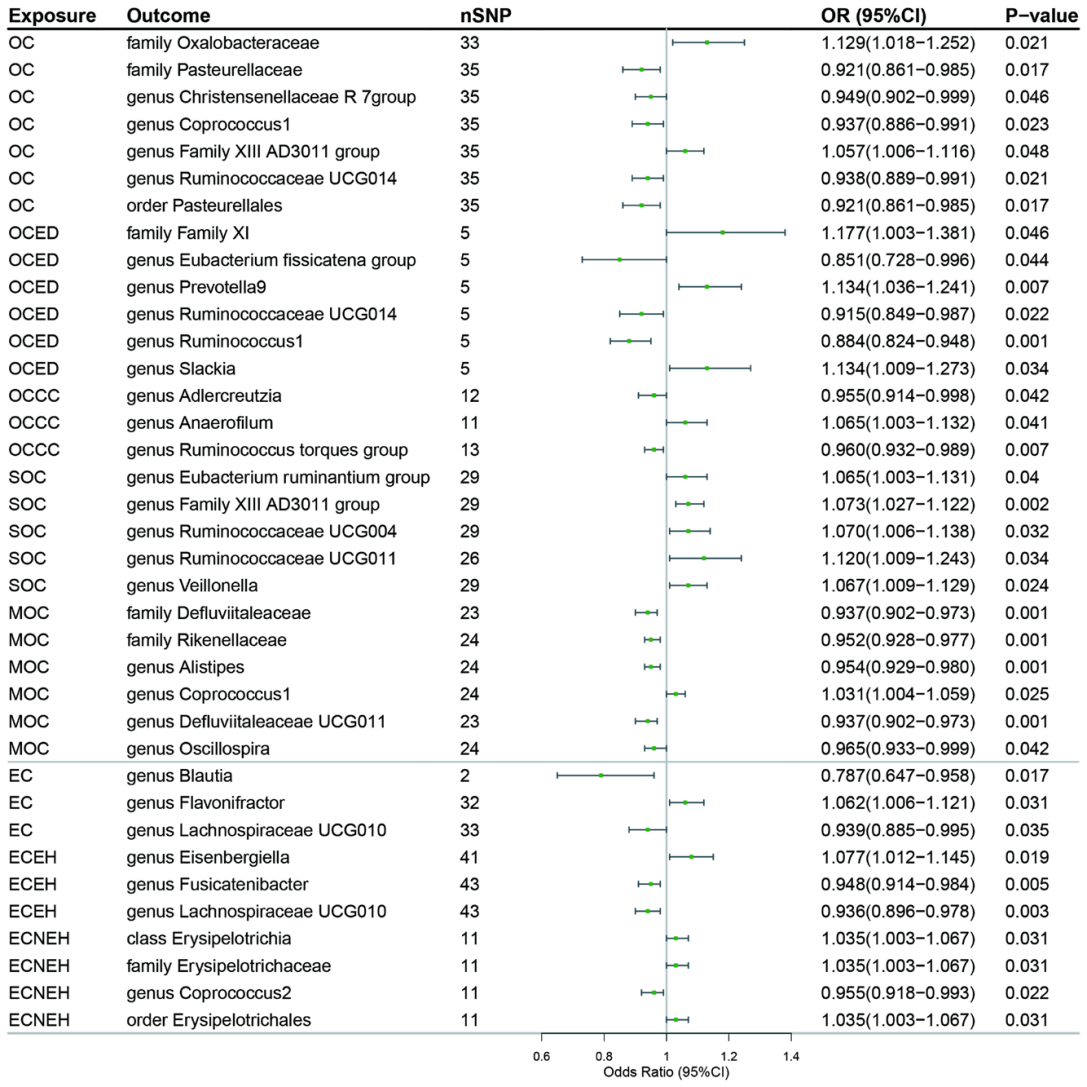
Forest plot of the association between OC, EC, and its subtypes and gut microbiome based on the IVW MR model. CI = confidence interval; EC = endometrial cancer; ECEH = endometrial cancer endometrioid histology; ECNEH = endometrial cancer non-endometrioid histology; HGSOC = high grade serous ovarian cancer; IMOC = invasive mucinous ovarian cancer; LGSOC = low grade serous ovarian cancer; LMPMOC = low malignant potential mucinous ovarian cancer; OC = ovarian cancer; OCCC = clear cell ovarian cancer; OCED = endometrioid ovarian cancer; OR = odds ratio; SNPs = single nucleotide polymorphisms; SOC = serous ovarian cancer.

To examine potential reverse causal effects, we executed an MR analysis using 1842 cancer-related SNPs as IVs (Table S4, Supplemental Digital Content, http://links.lww.com/MD/M6), with each gynecological cancer serving as the exposure and 196 gut microbiota taxa as the outcome. This analysis indicated that common gynecological cancers and their subtypes may exhibit causal relationships with 3 classes, 13 families, 41 genera, 4 orders, and 3 phyla species of gut microbiota (Table S5, Supplemental Digital Content, http://links.lww.com/MD/M7). We found that different cancers or different subtypes may affect the same microbiota. For instance, both OC and endometrioid ovarian cancer appeared to have a potential causal association with the *genus Ruminococcaceae UCG014*, whereas both LGSOC and IMOC were associated with the *order Verrucomicrobiales*. The results of the association between OC, EC, and gut microbiome are shown in Figure 5. In addition, all MR methods produced consistent direction of effect estimates. We found the statistics results showed no significant heterogeneity and no evidence of horizontal pleiotropy (Table S6, Supplemental Digital Content, http://links.lww.com/MD/M8).

**Figure 6.**
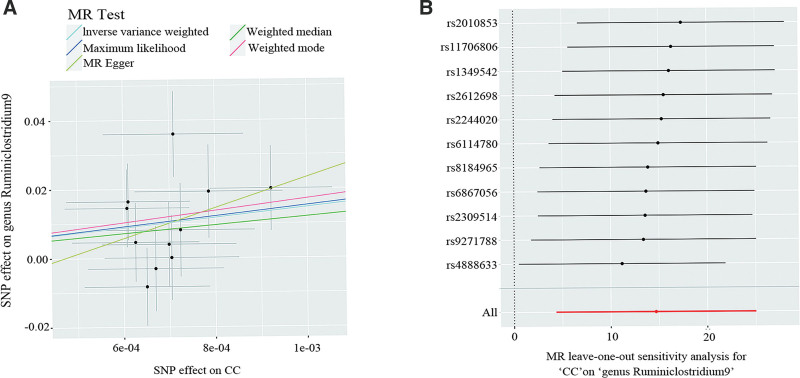
Reverse causal association between CC and the genus Ruminiclostridium9: scatter plot (A) and leave-one-out sensitivity analysis (B). Annotation: (A) Scatter plots of the CC-SNP associations (*x*-axis) versus the genus Ruminiclostridium9-SNP associations (*y*-axis) were shown, with horizontal and vertical lines showing 95% confidence intervals for each association. The lines that move obliquely upward from left to right show a positive causal direction between the taxa. (B) Leave-one-out analysis of the causal effect of CC on genus Ruminiclostridium9. The black dots represent the causal estimate of the association between a specific exposure and target after discarding each SNP in turn. Red dots represent the overall causal estimate using the random-effects IVW. Horizontal lines denote 95% confidence intervals. CC = cervical cancer; MR = Mendelian randomization; SNPs = single nucleotide polymorphisms.

Furthermore, we found a significant causal link between CC and the *genus Ruminiclostridium9 (P* = 5.381 × 10^−3^, IVW) (Fig. 6), suggesting a bidirectional causal influence between the 2. Nevertheless, we found no evidence of similar bidirectional causal relationships between other gynecological cancers and distinct gut microbiota.

## 4. Discussion

This research investigates the potential causal connections, between gut microbiota and 3 prevalent gynecological cancers: OC, EC, and CC, utilizing MR analyses. Our findings indicate that 33 species out of the 196 screened bacterial taxa, exhibit a suggestive causal link with the aforementioned gynecological cancers. Through an analysis of OR values, we identified 11 gut microbiota as potential risk factors, 19 as potential cancer risk reducers, and 3 as possibly protective against OC or EC, yet promotive in the context of CC. This suggests that gut microbiota plays a regulatory role in gynecological oncogenesis. To our knowledge, this is the first study to apply MR bidirectional analysis to evaluate the risk of gut flora in relation to gynecological cancers. We expect our findings may provide ideas for potential therapeutic targets related to gut flora and gynecologic cancers, with implications for public health interventions to reduce the risk of gynecological cancer.

In our findings pertaining to ovarian cancer, the *genus Peptococcus* emerged as a risk factor. A study proves that *Peptococcus* magnus is frequently isolated from significant infections,^[40]^ as a potential pathogen in formulating therapy for intra-abdominal sepsis, and severe infections of the skin. Numerous studies and epidemiological data underscore chronic inflammation’s role in ovarian carcinogenesis.^[41,42]^ It is widely recognized that specific gut microbiota can modulate immune and metabolic pathways, subsequently causing chronic inflammation.^[13]^ In a study by Lixing Chen,^[43]^ BPRN mice, induced with high-grade plasmacytoma of the fallopian tube and treated with antibiotics, displayed alterations in the composition of their gut and vaginal microbiota, which demonstrated antibiotics can alter the composition of tumor-associated microbiota, and that metabolites of these bacteria can promote immune responses, including responses to tumors. In this study, *Lanchnospiraceae* was identified as negatively associated with HGSC occurrence.^[43]^ This is the same result as the *genus Lanchnospiraceae Nc2004 groups* (OR = 0.76) in the conclusion of this study. Characterization of the associated flora within colon cancer tumor tissues after application of Immune checkpoint blockade therapy revealed that *Olsenella* produces a metabolite called inosine, which enhances the effects of checkpoint blockade immunotherapy, particularly targeting cytotoxic T lymphocyte-associated antigen 4.^[44]^ This result was similarly confirmed in bladder cancer, kidney cancer, and melanoma. In our results, *genus Olsenella* was similarly negatively associated with ovarian carcinogenesis with a protective effect. Based on the above viewpoints, the MR results of this study may provide broader ideas and directions for exploring the prevention of ovarian cancer by regulating the flora and further improving the immunotherapy of ovarian cancer.

Data show that the microbiome of ovarian tumors is completely different from its surrounding noncancerous tissues^[45]^ and has reduced diversity.^[46]^ A study of the identification of associated flora in ovarian cancer samples showed that *phylum Proteobacteria* (52%) and *phylum Firmicutes* (22%) were the predominant genera when 99 ovarian cancer samples were compared with tissues considered adjacent to the tumor by pathological analysis.^[45]^ In another study,^[47]^ *phylum Proteobacteria* increased and *phylum Firmicutes* and *phylum Bacteroidetes* decreased in the composition of the gastrointestinal microbiota of patients with lung cancer after treatment, and both of these studies are at variance with our results that both *Proteobacteria* and *Firmicutes* in the intestinal flora of patients with ovarian cancer are protective factors. However, Sagarika Banerjee^[45]^ found that *phylum Proteobacteria* was equally found in control benign samples. It is hypothesized that this may be related to 3 reasons. First, it is not clear how the human gut flora relates to the flora in ovarian cancer tissues, and it still needs to be investigated whether the findings are the same in both. Second, most studies have determined whether gut microbes are “beneficial” or “harmful” by comparing the relative abundance of gut microbiota in healthy individuals and patients,^[37]^ but mechanistic studies are needed to make reliable predictions about the role of gut biota in ovarian cancer development. In this process, data comparability and common standards, procedures, and methodologies are important, which need to be further confirmed by more studies. Finally, because the minimal classification of the MR study was only down to the genus, the lack of precision in describing the names of specific bacteria within the genus led to the discrepancy in results. In any case, however, it is not difficult to conclude that summarizing a well-defined microbiome profile of ovarian tumors can provide a way of detecting cancer occurrence or prognosis.

In addition, we analyzed a total of several different pathological types of ovarian cancer, with only a few duplicates of the gut microbiota, and it can be hypothesized that different genera are significantly associated with the clinicopathology and characteristics of patients with ovarian cancer. This was confirmed in a study by Jinfei Tong,^[48]^ who found that *genus Ruminococcus* and *genus Ruminococcaceae* were significantly associated with gastrointestinal reactions. In addition, *Bifidobacterium, Megamonas*, and *Pseudomonas* were significantly higher in patients with shorter survival periods, and *Klebsiella* and *Fusobacterium* were lower in patients with longer survival periods. However, relatively few studies have been conducted on different pathologic features, and further relevant studies can be added in the future.

The relationship between gut microbiota and endometrial cancer has been confirmed by numerous studies. Estrogen-encoded β-glucuronidase and β-glucuronide are able to act in the gut,^[49]^ where intestinal microbiota such as *Lactobacillus* remove estrogen-binding glucuronides via biliary excretion, acquire free estrogen molecules, and regulate circulating estrogen levels.^[50]^ Meanwhile, *Lactobacillus* was able to enhance the expression of genes associated with healthy intestinal permeability to regulate intestinal microecological stability, restore intestinal barrier function,^[51]^ and potentially modulate body immunity by activating or inhibiting cytokine expression.^[52]^ This is consistent with our findings that *genus Lactobacillus* may be a potential protective factor for EC, suggesting that it may reduce the risk of EC development by regulating estrogen levels and immune function.

A prospective case-control study found that patients with EC have a high abundance of *Prevotella*, which is a crucial factor linked with cancer burden.^[53]^ This is not consistent with our findings of *Paraprevotella* as a protective factor for ECEH in the current study. In addition, no solid evidence was discovered in the reverse MR analysis that EC affects the properties of *Paraprevotella*. This may require further studies for verification. *Lactobacillus*, by producing lactic acid, aids in sustaining the low pH of the reproductive tract, serving as a selective barrier to hinder the colonization of opportunistic pathogens like *Clostridium* and *Escherichia coli*, thereby contributing to the stability of the microbiota.^[54]^ This aligns with our research conclusion that *Lactobacilli* can serve as a potentially protective factor against EC, with hopes of being used for clinical adjuvant therapy and further research validation.

Patients with CC also exhibit gut microbiota dysbiosis.^[55]^ Early-stage CC patients have higher levels of *Prevotella* compared to the healthy control group.^[56]^ This aligns with our research findings: in the MR analysis, we observed that *Prevotella9* may increase the risk of CC. The reverse MR results demonstrated that CC had no causal effect on *Prevotella9*, suggesting that the proliferation of *Prevotella* may not be induced by CC. Moreover, *Prevotella* levels are also elevated in patients with late-stage CC.^[57]^ We postulate that *Prevotella9*, sustaining elevated levels in CC, might serve an estrogenic or immunomodulatory function.

Our findings revealed significant differences in the bidirectional MR analysis between *genus Ruminiclostridium9* and CC. Alterations in *Ruminiclostridium* could impact the host’s immune system or inflammatory response, influencing the risk of CC, and CC or its treatment may also change the gut microenvironment, thereby affecting the composition of *Ruminiclostridium*. Additionally, our findings indicate that the causal effect of the gut flora on CC is not significant. It is surmised that the primary etiology of CC is persistent HPV infection, intricately linked with the microenvironment of the lower reproductive tract.^[13]^ The large OR and *Beta* values in the reverse MR analysis of CC may imply a large effect of genetic variants on CC, thus affecting the gut microbiota, but the large *SE* and 95% CI values imply a large uncertainty in the sample means, possibly due to insufficient sample size or a large variability in the sample distribution. Unfortunately, the current impact of the gut microbiota on the vaginal microenvironment remains ambiguous, and there are too many influencing factors that confounding errors cannot be ruled out. Further research is required to elucidate the connection mechanism between these 2 elements to clarify the reasons for the above issues.

Our reverse MR analyses indicate that cancer also affects alterations in the gut microbiota. Indeed, some therapeutic modalities can also affect cancer by targeting the gut flora.^[58]^ Compared with preoperative fecal samples, the abundance of *Bacteroidetes* and *Firmicutes* in postoperative fecal samples significantly decreased, while the abundance of *Proteobacteria* significantly increased.^[48]^ Besides, extensive research shows that gut microbiota can modulate the metabolism of chemotherapy drugs, which affects the response and sensitivity to chemotherapy in cancer.^[59,60]^ Therefore, the gut microbiota not only affects the occurrence and progression of cancer but also is affected by cancer treatment and can influence the therapeutic effects. In the future, further clarification of the strategies and effectiveness of applying gut microbiota to treat cancer may provide new insights for cancer treatment. Considering the intricate relationship among treatment modalities, gut microbiota, and cancer,^[61]^ more research and mediator MR analyses are necessary to elucidate their associations and mechanisms.

Geographic and ethnic differences (including genetic, environmental, dietary, environmental, ethnic lifestyle, and religious) all affect the composition and characterization of gut flora among individuals and groups. The geographic origin of a population has a greater impact on the composition of the gut microbiota than BMI or gender.^[62]^ Significant differences in gut flora were found between Asian, African, and European races,^[63]^ with European races dominated by *phyla Firmicutes, Bacteroidota, Actinobacteria, Proteobacteria, Fusobacteria*, and *Verrucomicrobia*, which may be more associated with protein or fat metabolism.^[64]^ The gut flora of Asians may be richer in certain bacteria associated with carbohydrate metabolism, such as *Prevotella, Bacteroides, Lactobacillus, Faecalibacterium, Ruminococcus, Subdoligranulum*, and *Coprococcus*.^[65,66]^ African races may tend to possess microbial species associated with dietary fiber breakdown, such as *Bacteroides* and *Prevotella*.^[67]^ These findings emphasize the need to consider ethnic background when studying and applying gut flora. However, most of the current studies have focused on Europeans or Americans, with limited understanding of the gut microbiome in other regions and ethnicities, so more large-sample gut flora studies are expected to enrich the gap in this area to create a database of independent genomic association studies across regions and ethnicities.

Nevertheless, this study shares similar limitations with most MR studies currently conducted. Firstly, current studies lack uniform methods and standards for the measurement of gut microbiota, with considerable variation in sample extraction management. The differences in sequencing platforms and research levels of the analysis process could result in discrepancies and incomparabilities in the outcomes. Secondly, to avoid the heterogeneity of results and the multi-effects of instrumental variables, the study included mainly individuals of European ancestry, which may limit the extrapolation of our findings to other races. In addition, our study did not cover all gynecologic tumor types due to the sample size and type limitations of the GWAS database used. However, our study has several advantages. Firstly, we conducted a study of different pathologic types of gynecologic tumors to find the differences. Secondly, the included patients are not affected by the confounding factor of gender, on this basis, the results may be more reliable when the identified causal relationship is confirmed. Additionally, regarding the application of instruments, a comprehensive GWAS meta-analysis is utilized to obtain the genetic variation of the gut microbiota, ensuring the dependability of the analysis tool.

Moving forward, conducting detailed mechanistic studies is essential to precisely determine the role of specific gut microbes in the development of gynecologic cancers. This includes exploring their metabolite secretion, impact on host immune responses, and direct interactions with host cells using in vitro experiments, animal models, and preclinical studies. Standardizing gut microbiome measurements will reduce variability caused by different technological approaches. Further, encouraging interdisciplinary collaborations will accelerate the exploration of complex interactions between gut microbes and gynecologic tumors, leading to innovative preventive and therapeutic strategies based on gut microbiome alterations.

## 5. Conclusion

Our study summarizes some causal effects of gut microbiota on 3 gynecological cancers from a genetic analysis, 11 of these genera could be pathogenic risk factors, while 19 could lessen the risk of cancer. In the other direction, gynecological cancers also altered gut microbiota composition. The beneficial or harmful gut microbiota pinpointed in this study could furnish valuable insights into the pathogenic mechanisms of the above-mentioned 3 gynecological cancers mediated by the microbiota and strategies aimed at their prevention and therapy.

## Acknowledgments

This study was possible thanks to the participants of all GWAS cohorts included in the present work and IEU Open GWAS project, MiBioGen, UK Biobank, OCAC, MRC-IEU, ECAC, and E2C2 for sharing the GWAS summary statistics.

## Author contributions

**Conceptualization:** Youqian Kong.

**Data curation:** Youqian Kong.

**Methodology:** Youqian Kong.

**Project administration:** Rui Qie.

**Software:** Youqian Kong.

**Supervision:** Rui Qie.

**Visualization:** Youqian Kong, Shaoxuan Liu.

**Writing – original draft:** Youqian Kong, Shaoxuan Liu, Xiaoyu Wang.

**Writing – review & editing:** Youqian Kong, Shaoxuan Liu, Rui Qie.

## Supplementary Material














