# Supplementary material for: Associations between gut microbiota and gynecological cancers: A bi-directional two-sample Mendelian randomization study
Source: Medicine (Baltimore). 2024 Mar 29;103(13):e37628. doi: 10.1097/MD.0000000000037628 (PMC10977594; doi:10.1097/MD.0000000000037628)

**Supplementary Figure S1. Forest plots for the Mendelian randomization (MR) leave one out analysis of the significant and nominal significant results.**

Within each panel, the black points represent the causal estimate of association between a specific exposure and target gynecological cancers after discarding each SNP in turn. Red points represent the overall causal estimate using the random-effects inverse variance weighted. Horizontal lines denote 95% confidence intervals.

**1. Ovarian cancer**


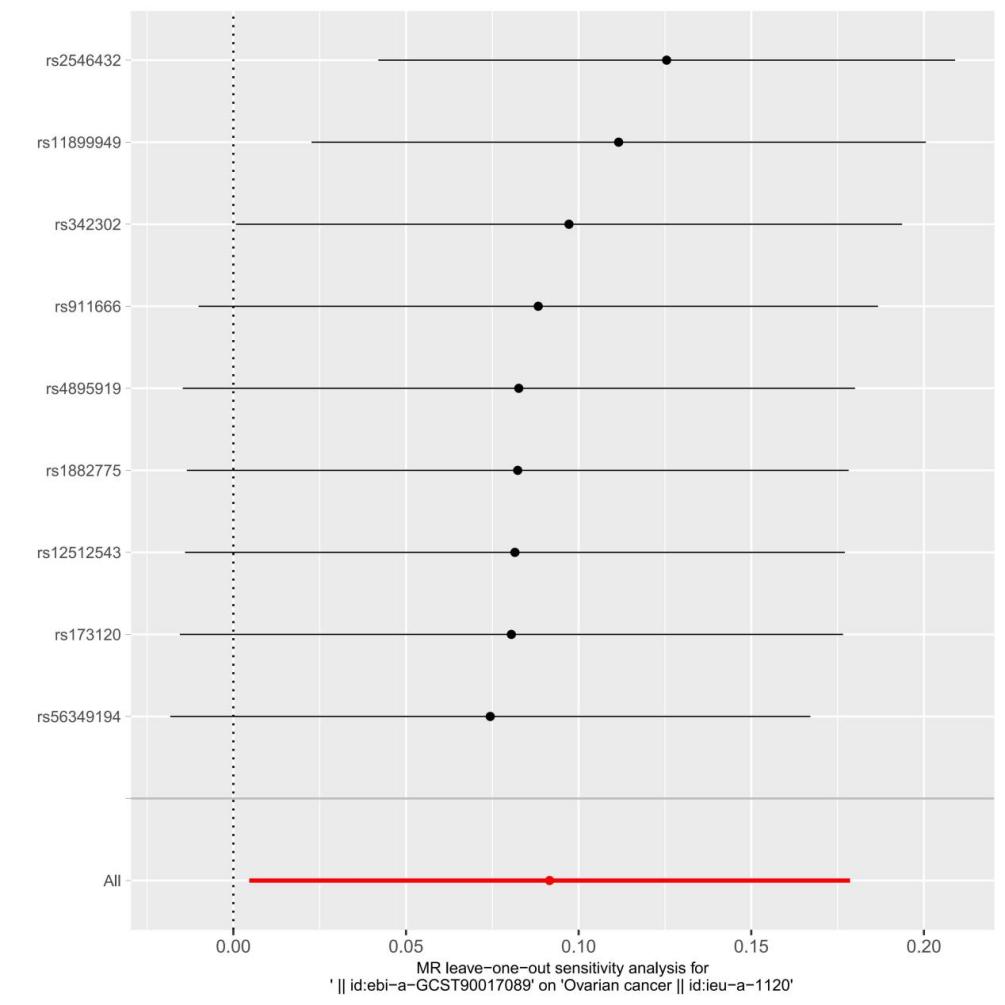

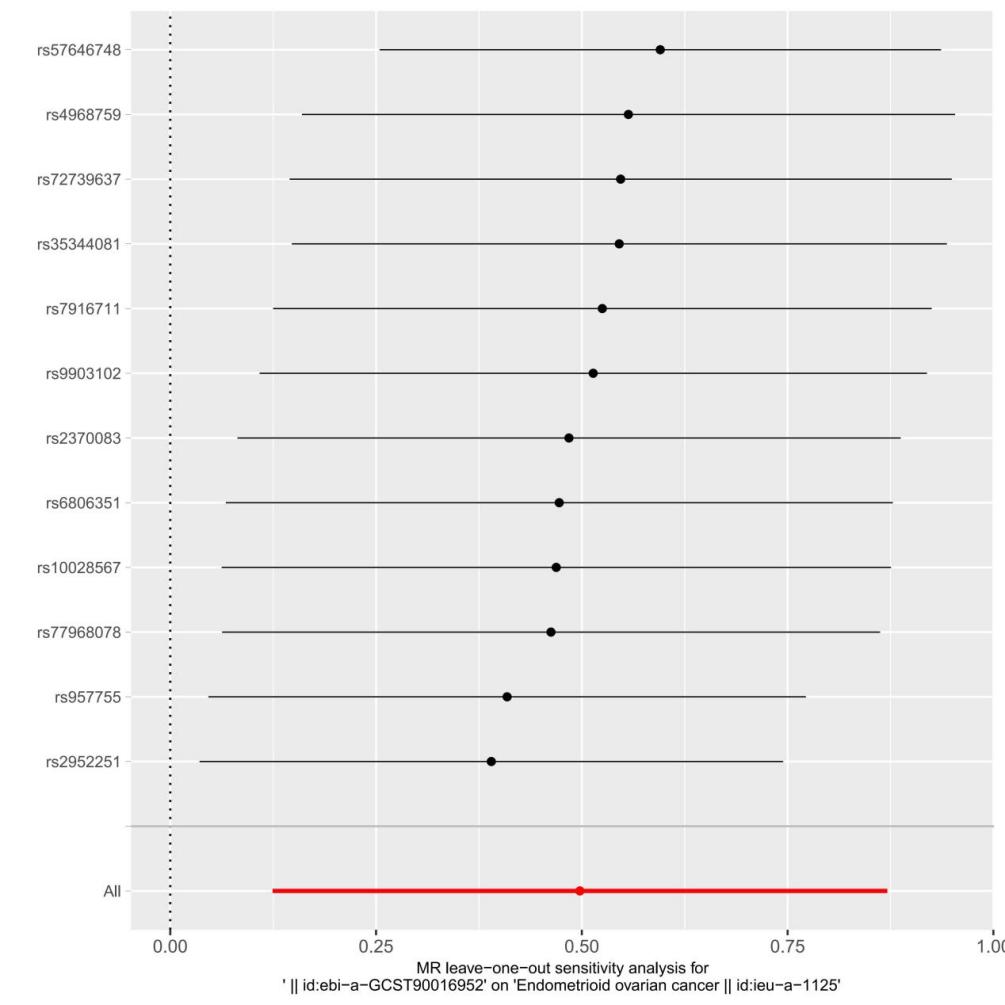


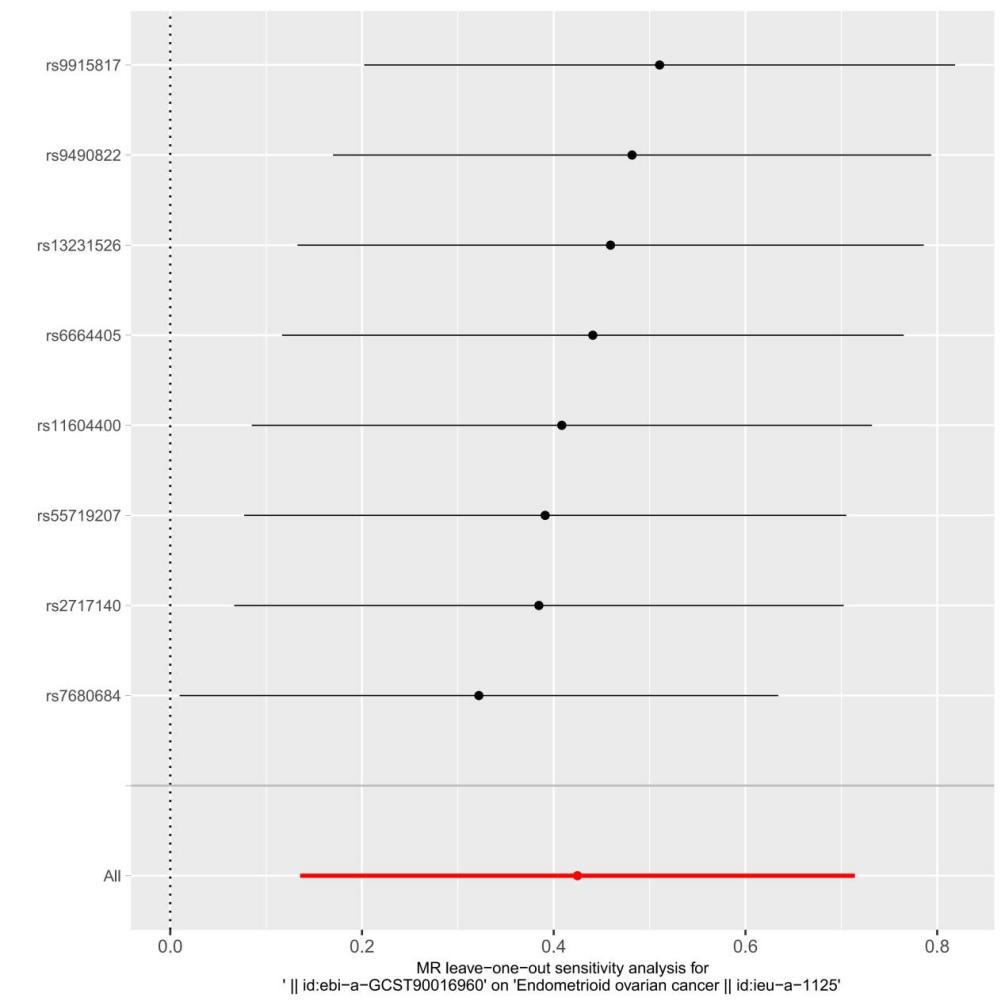

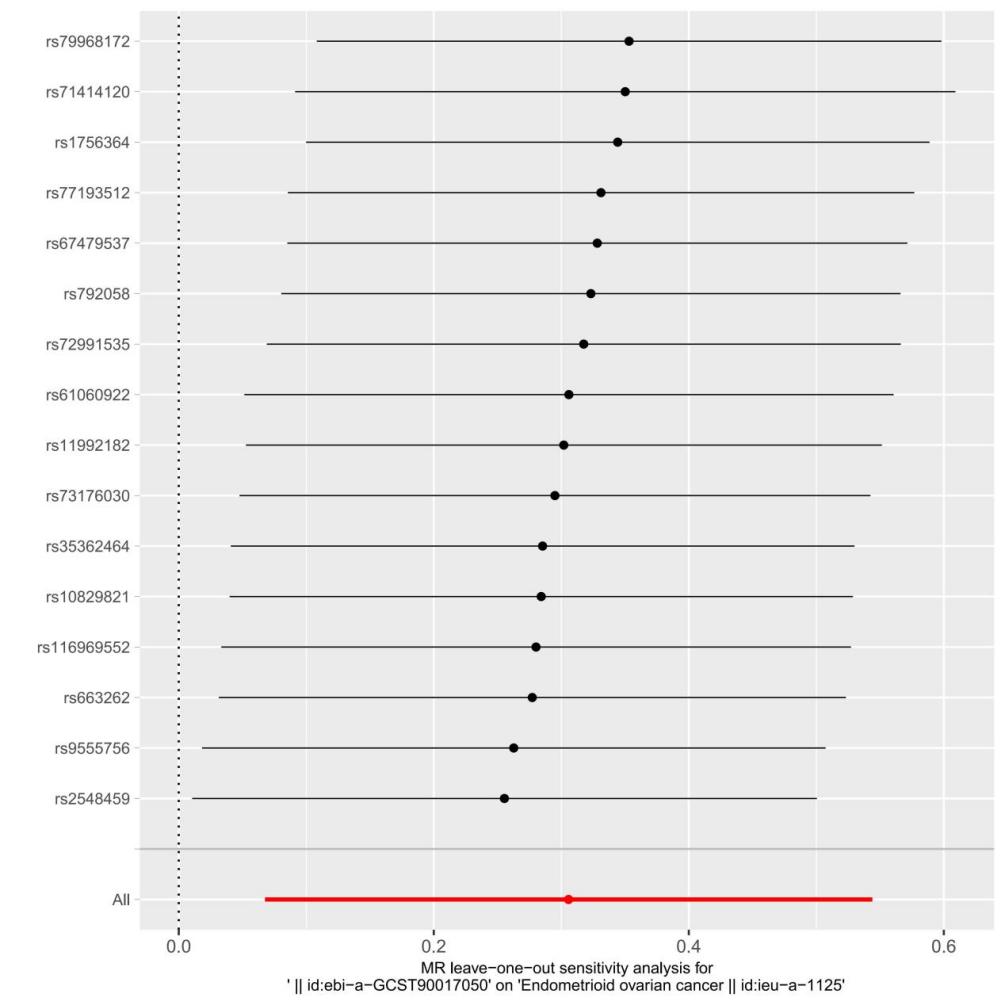

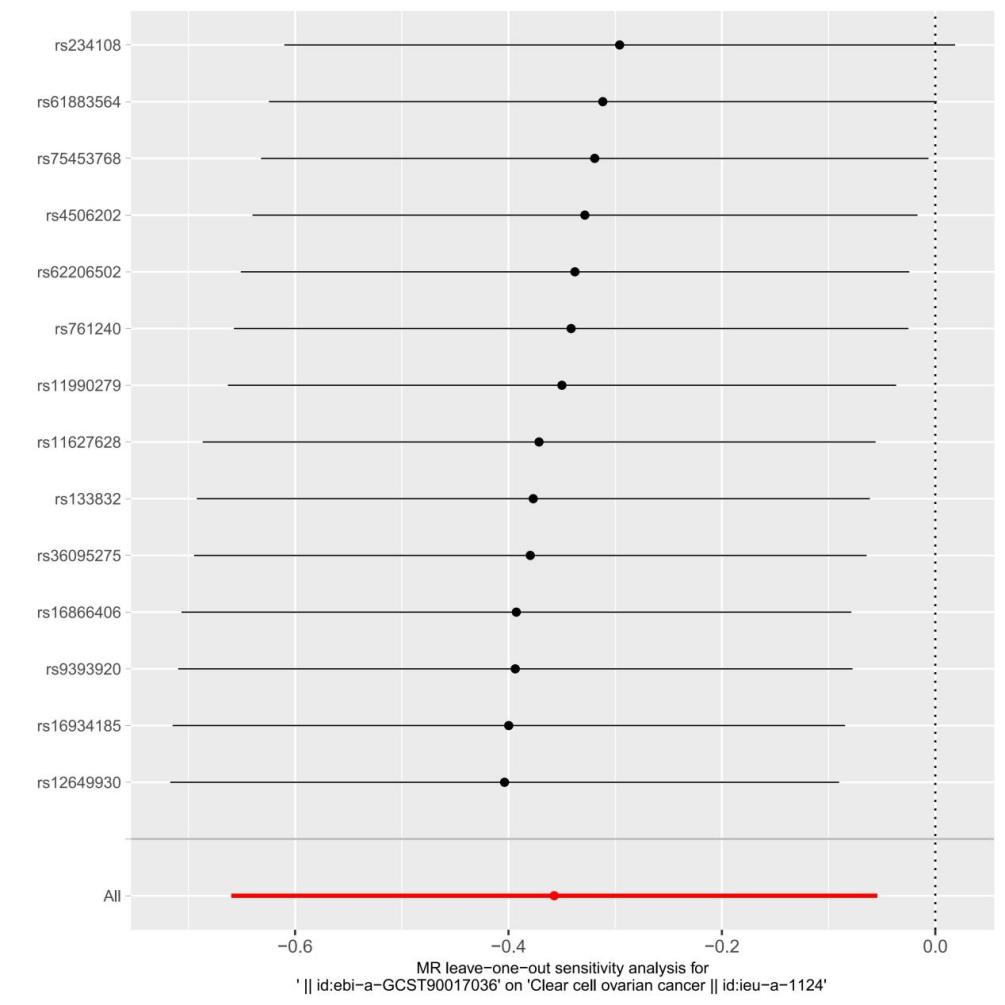

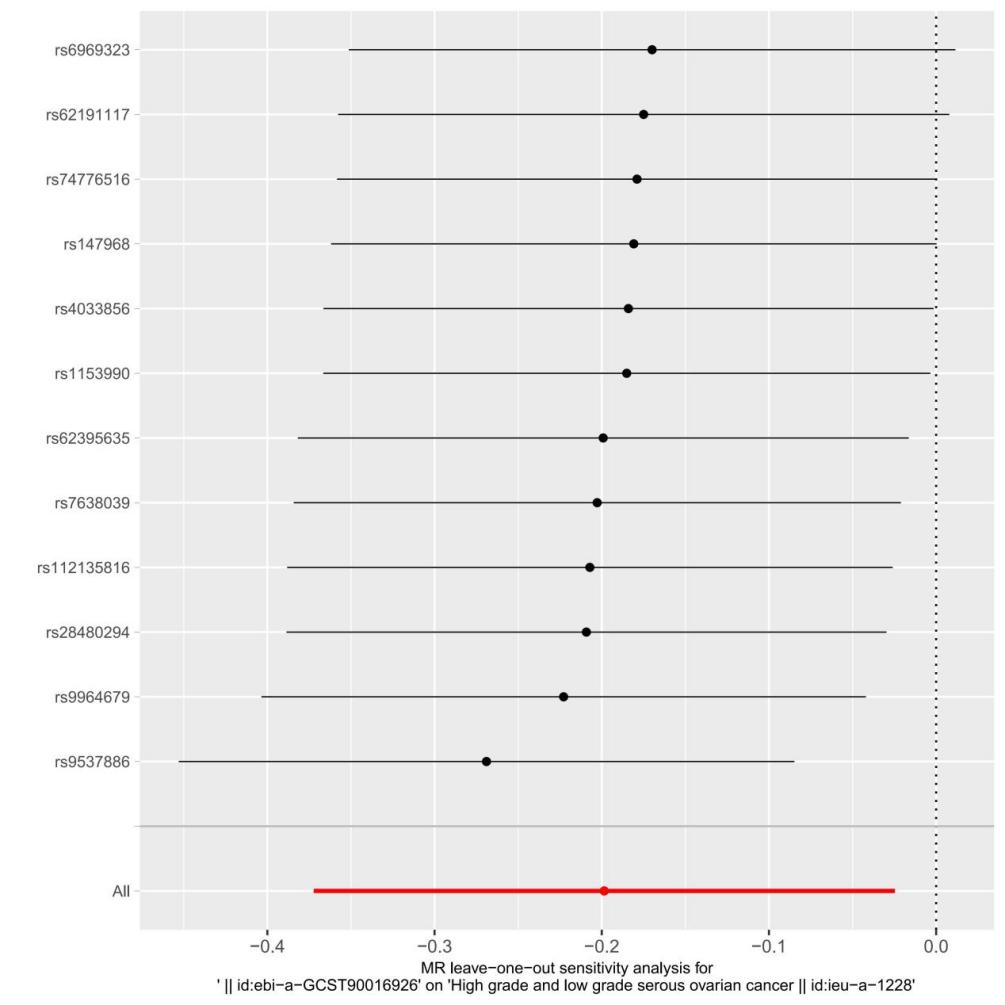

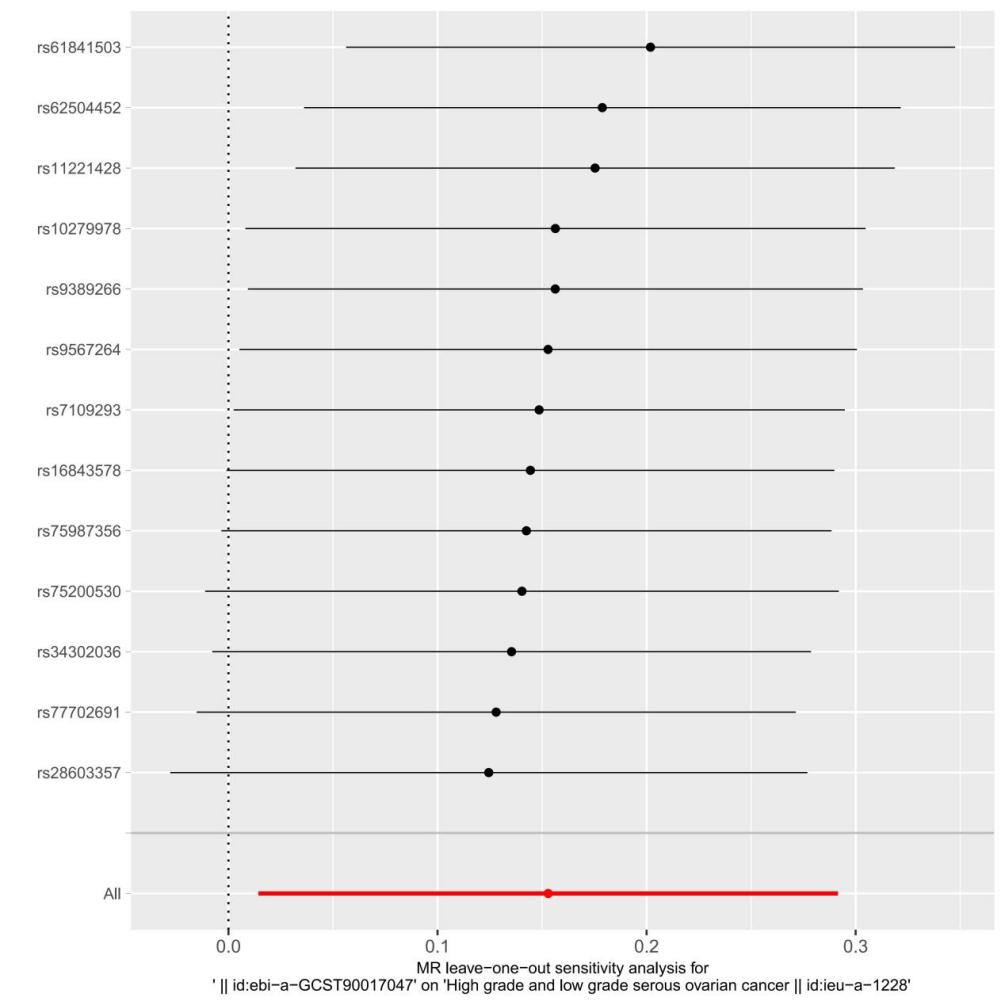

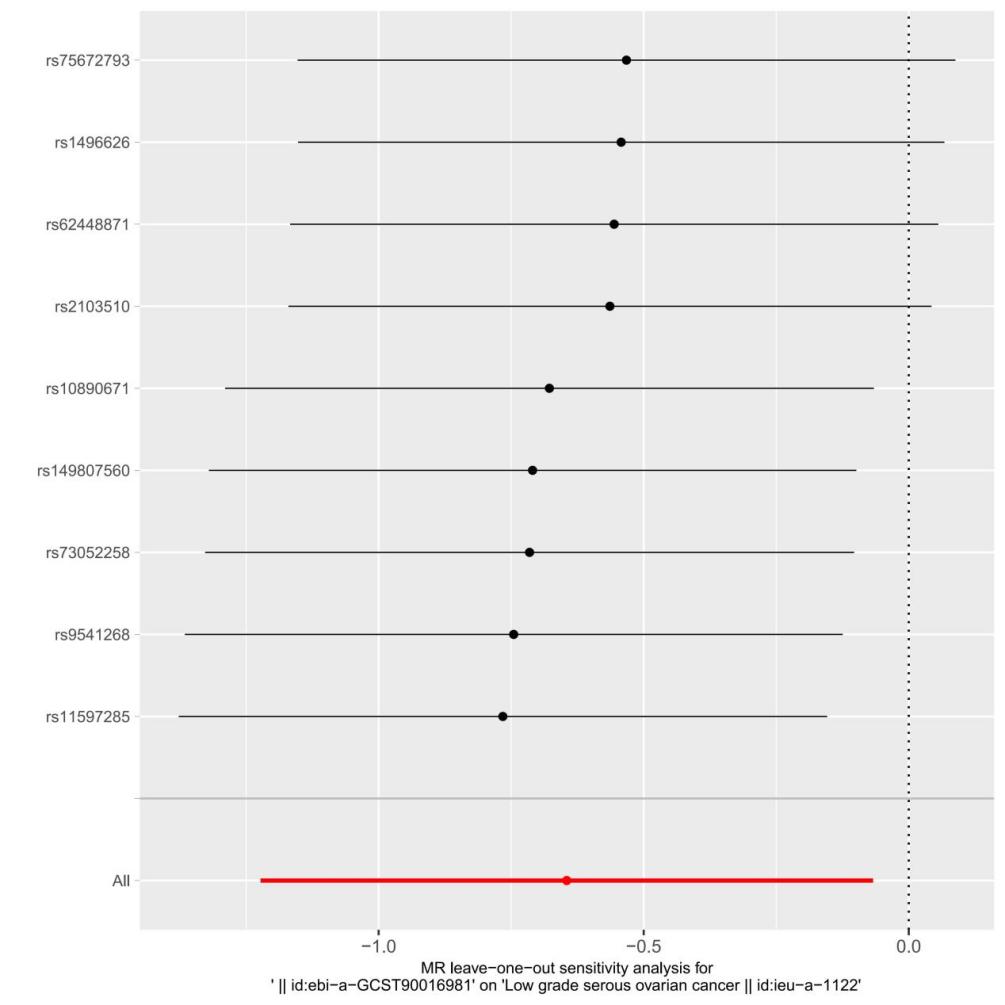

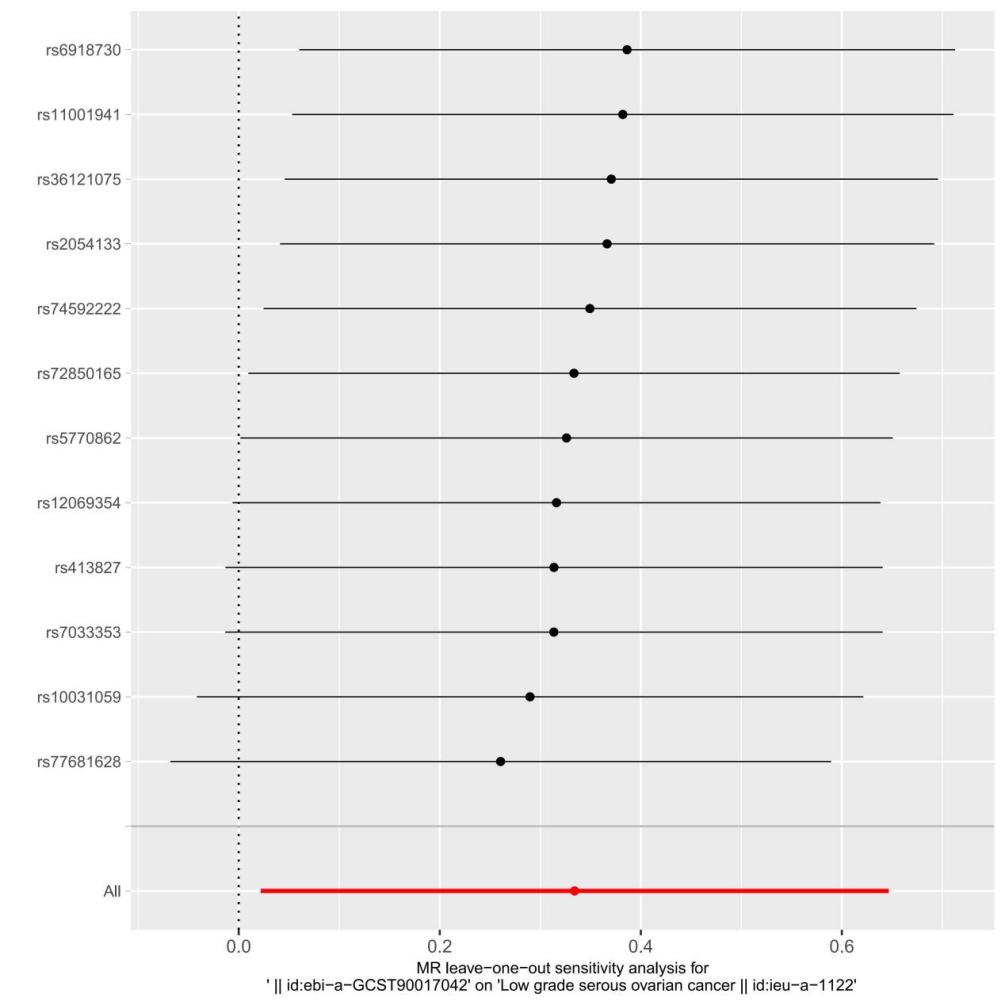

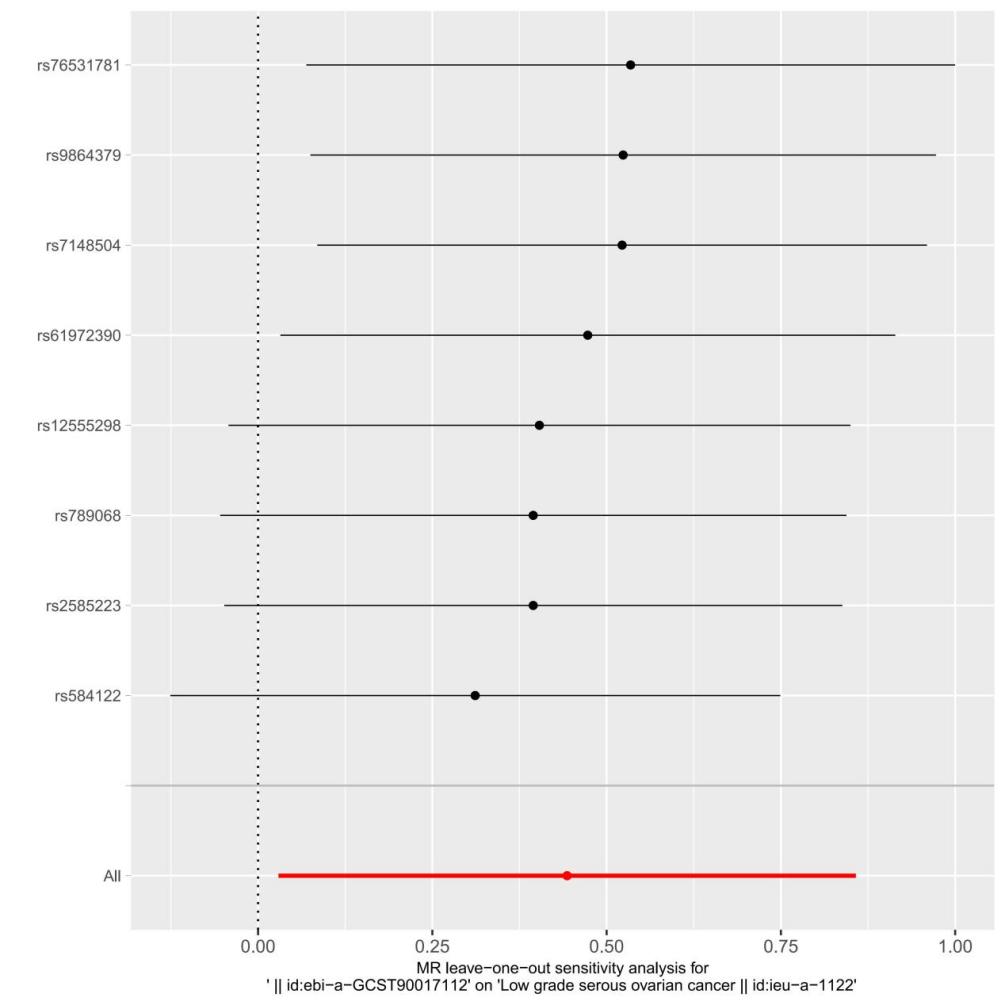

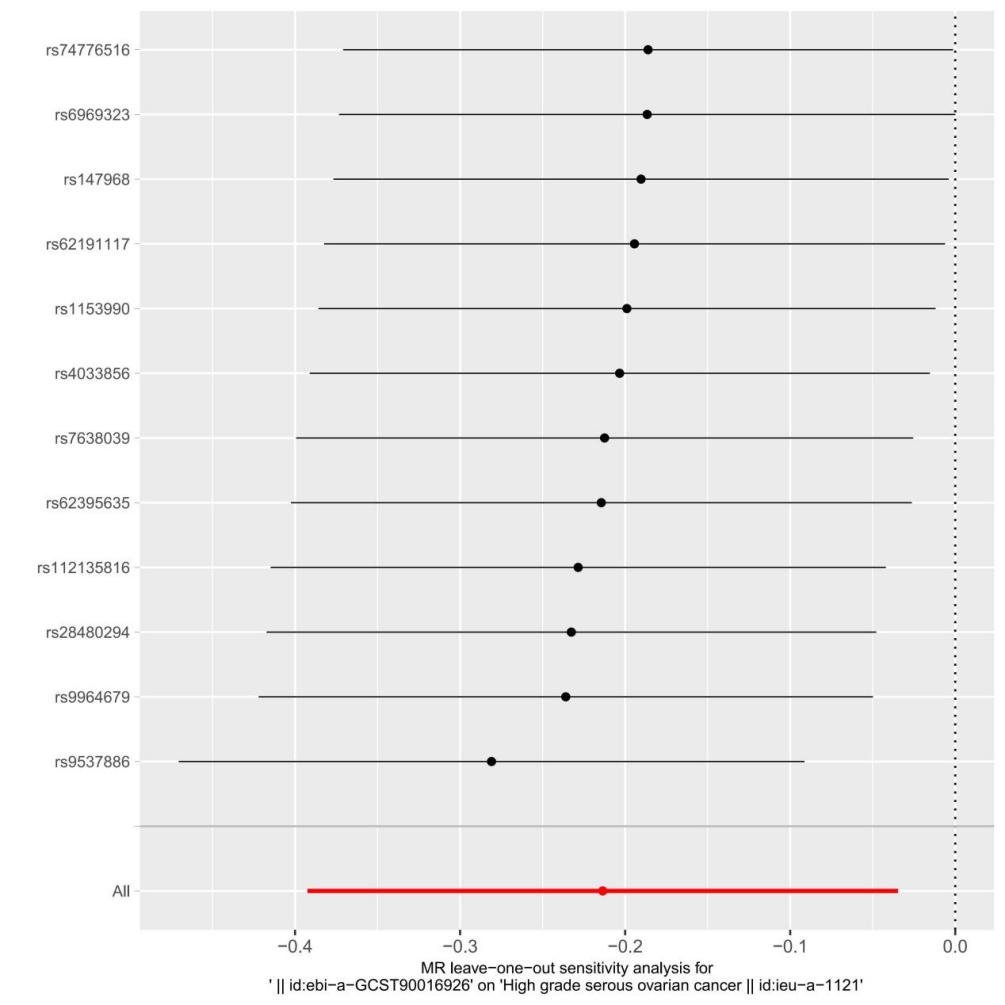

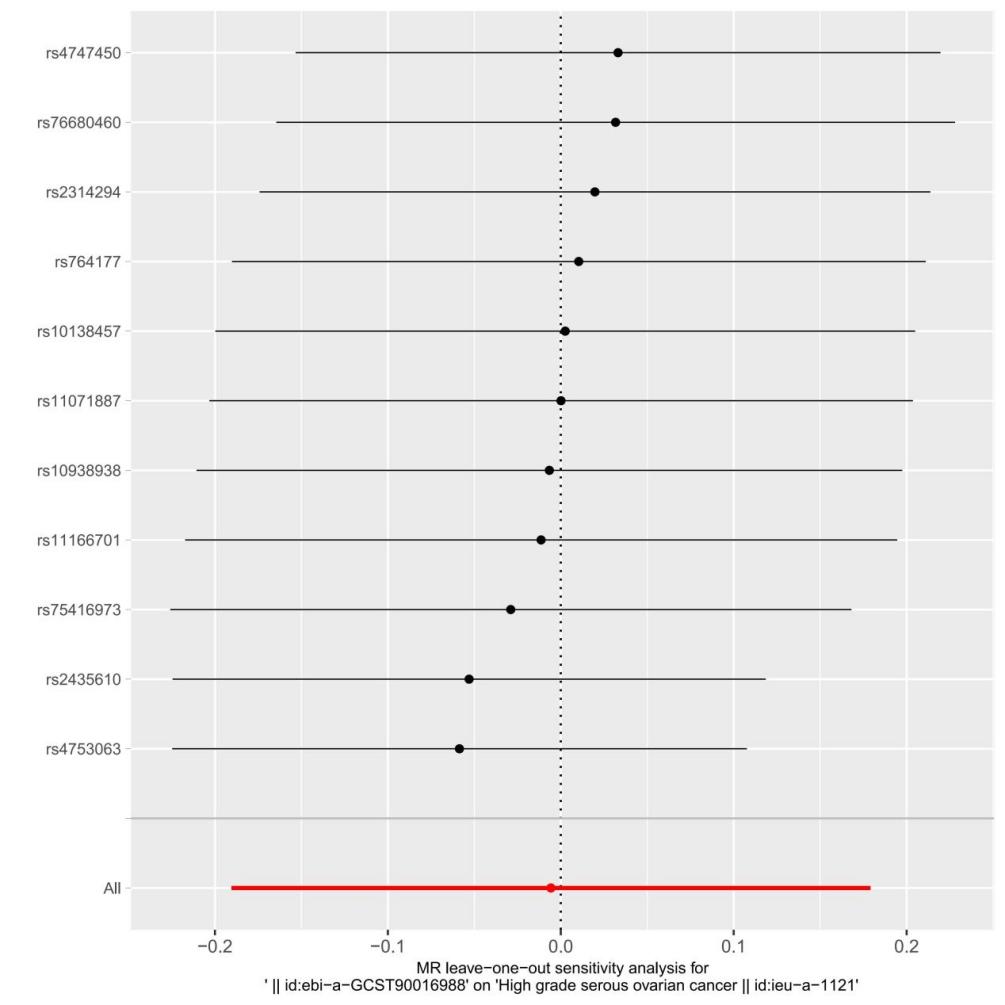

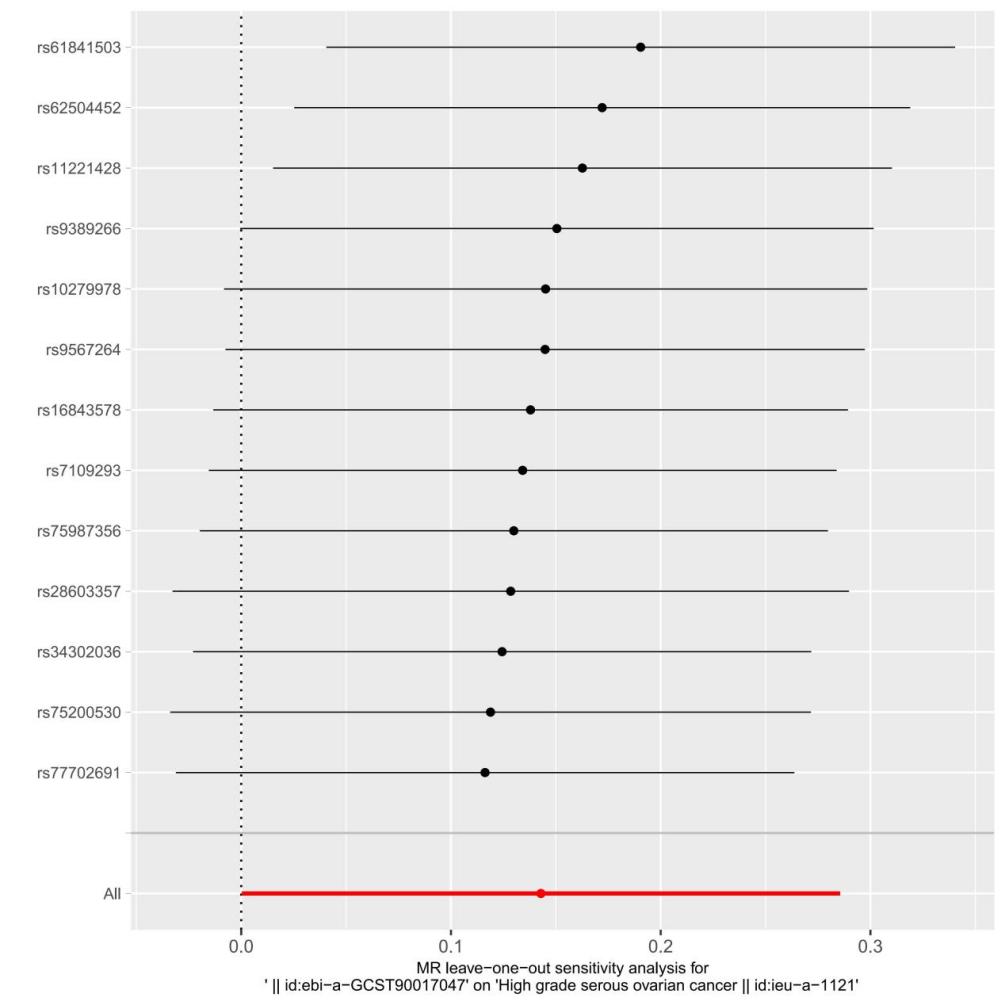

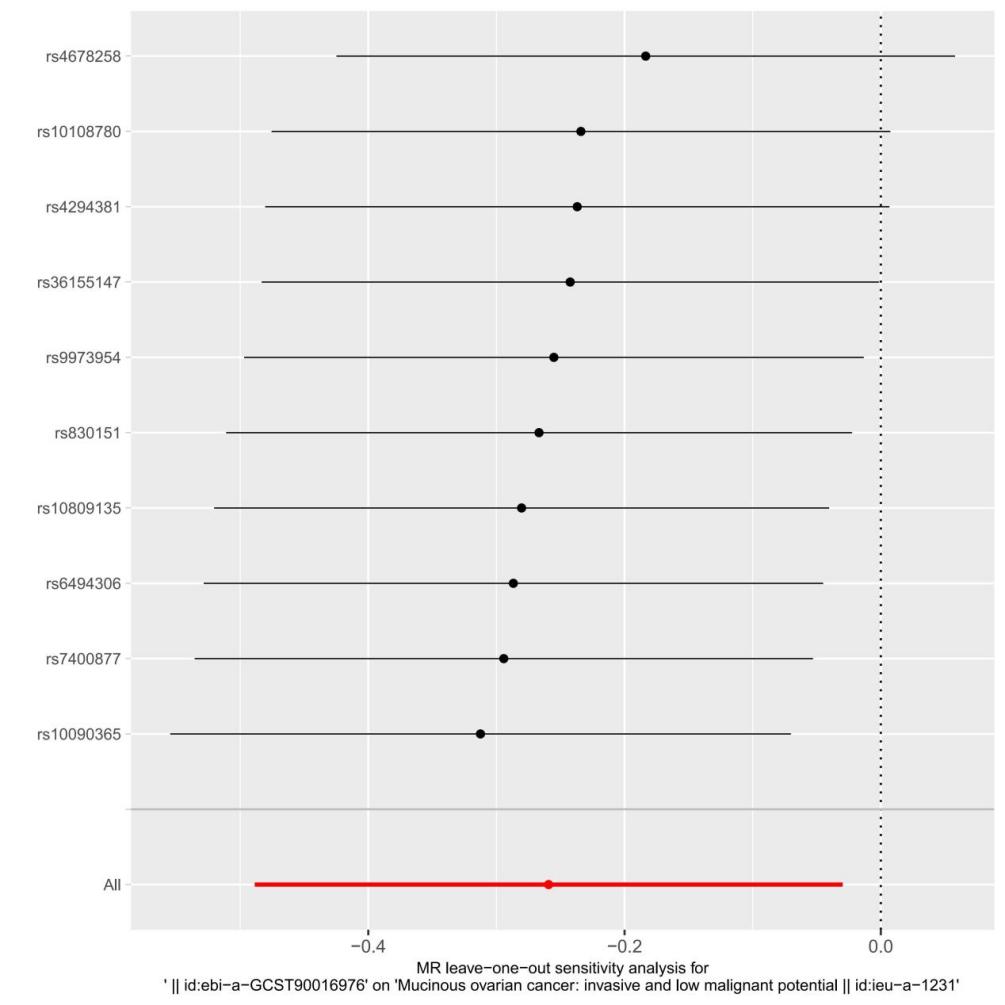

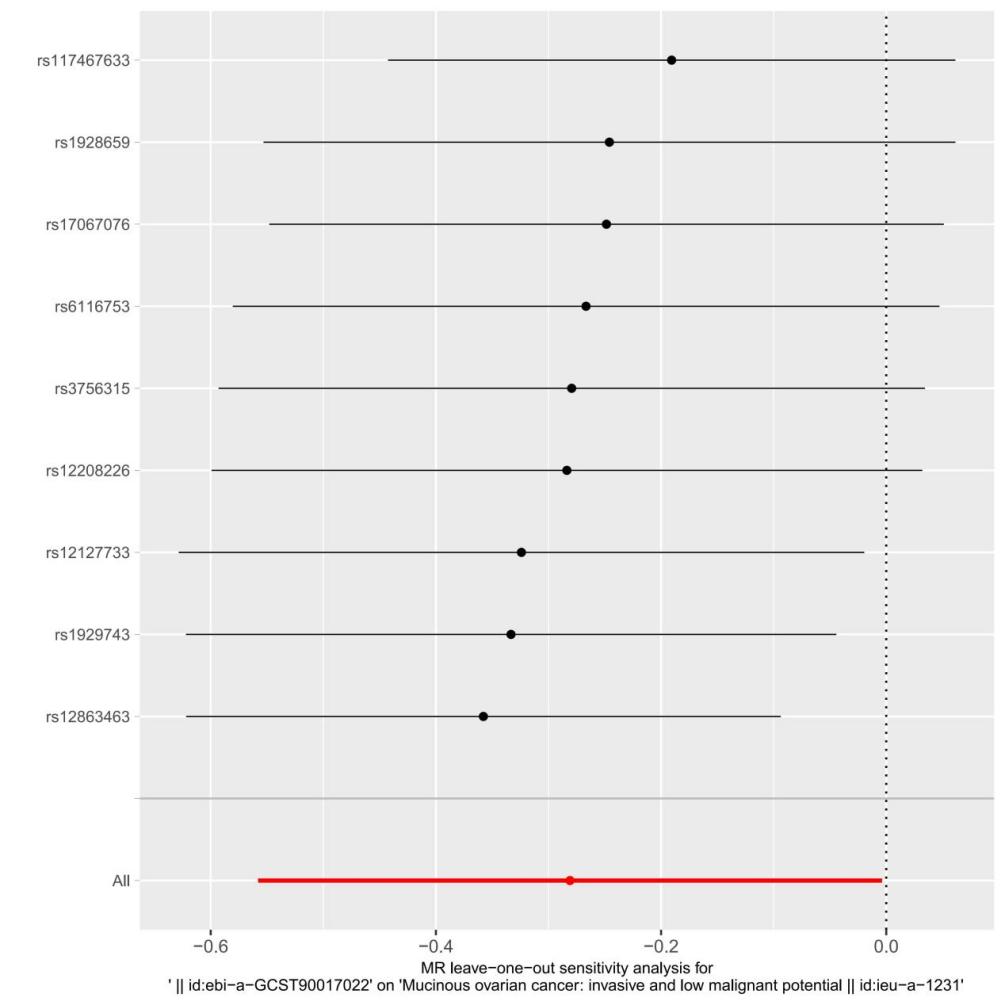

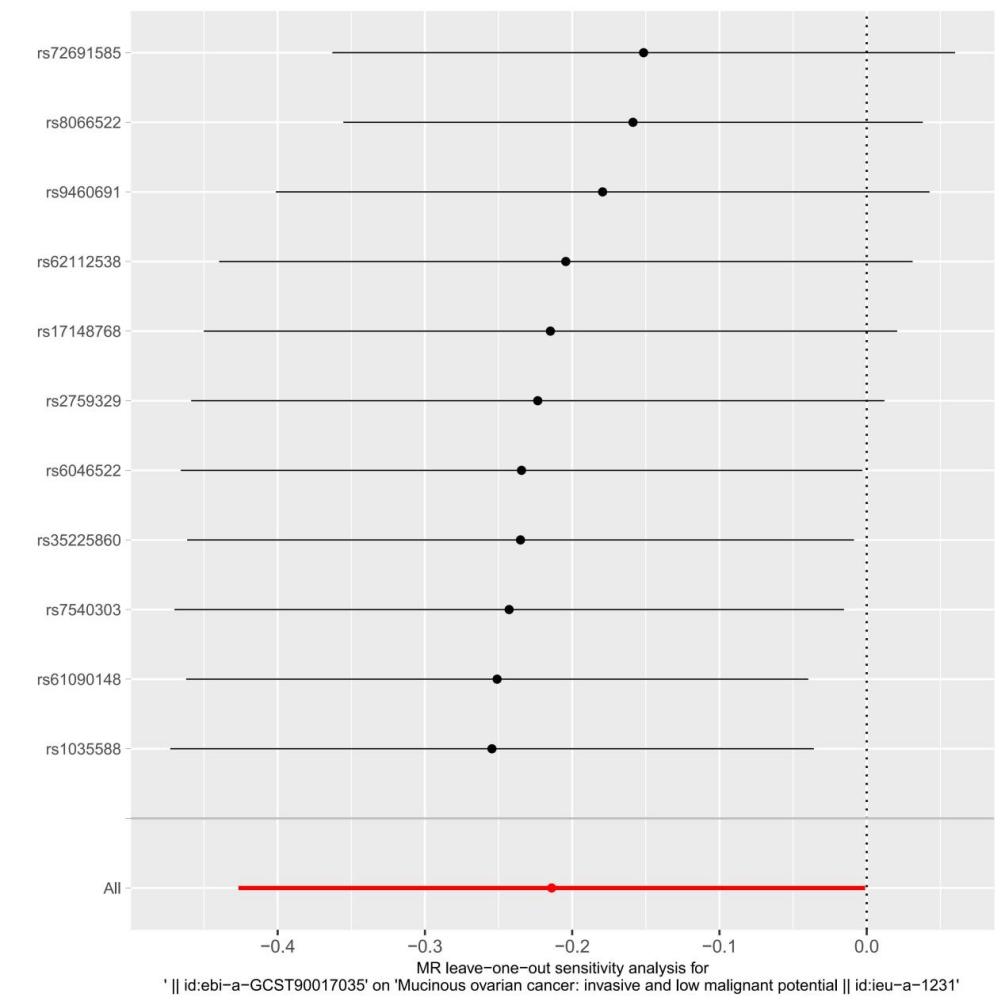

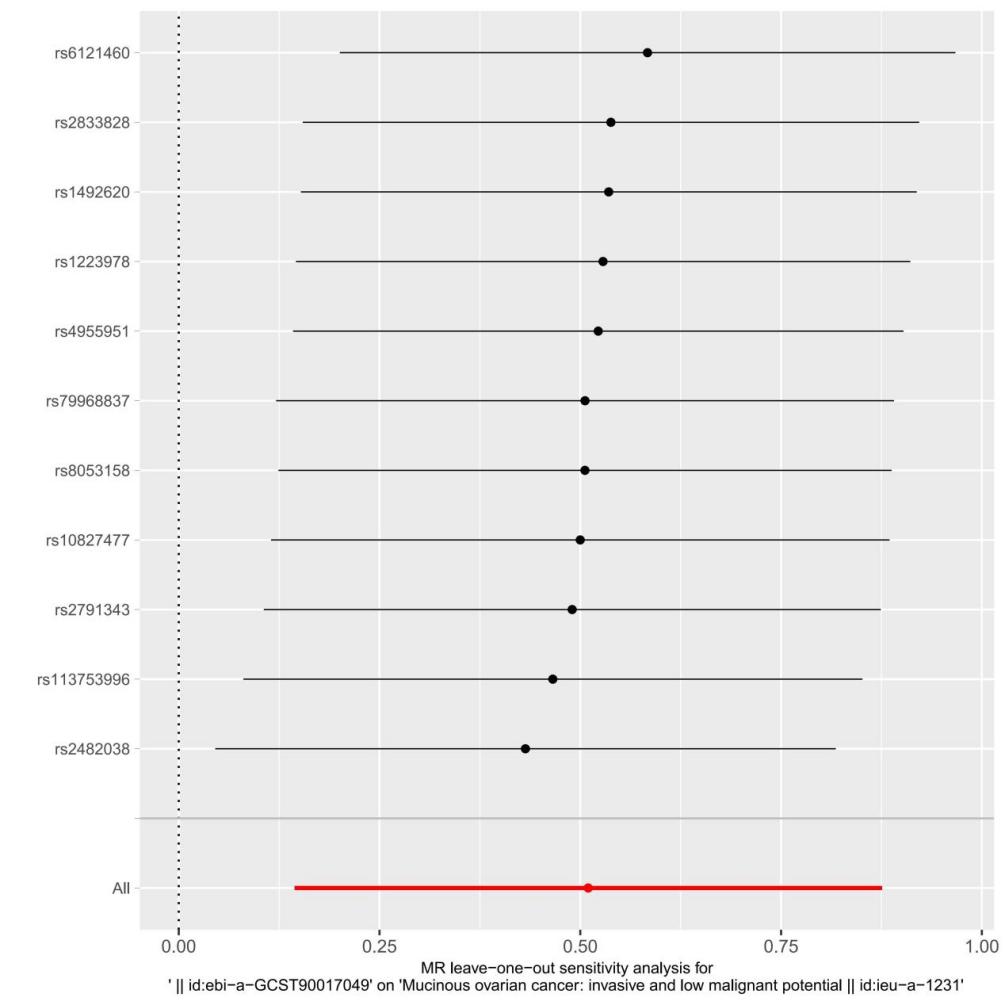

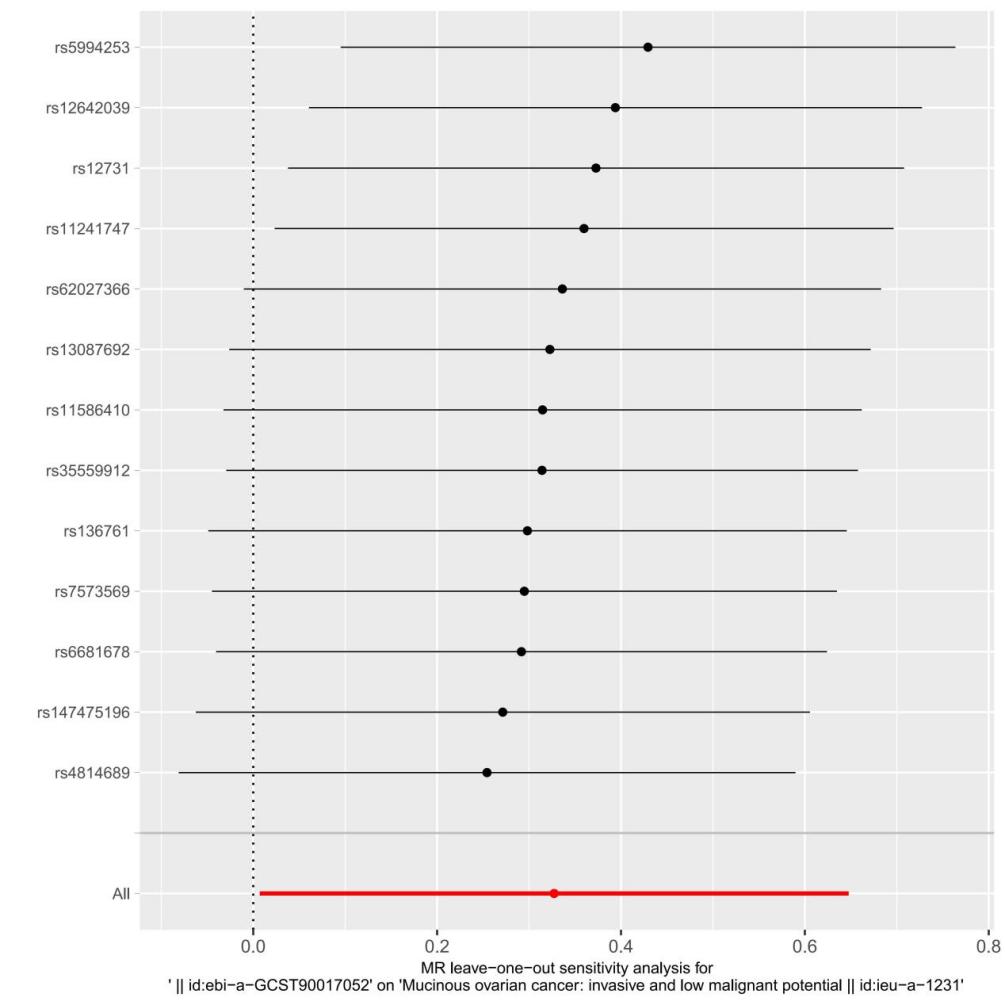

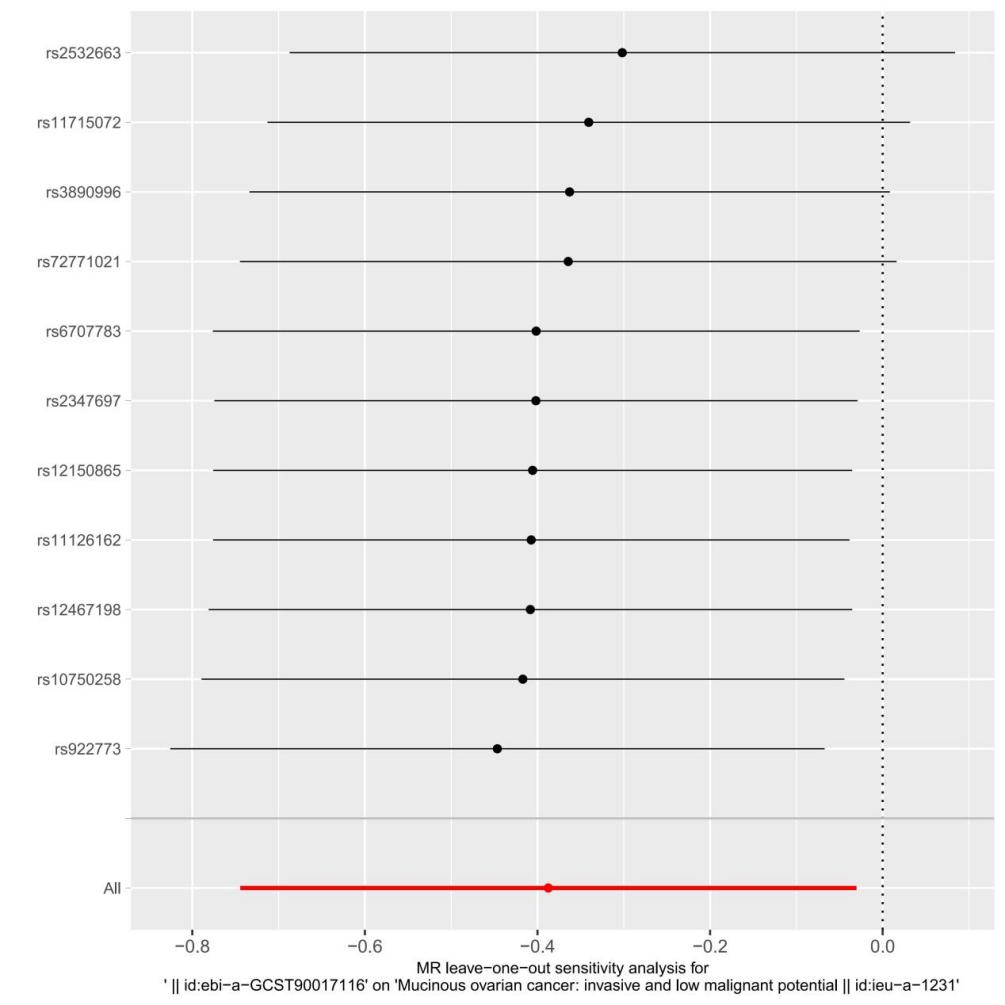

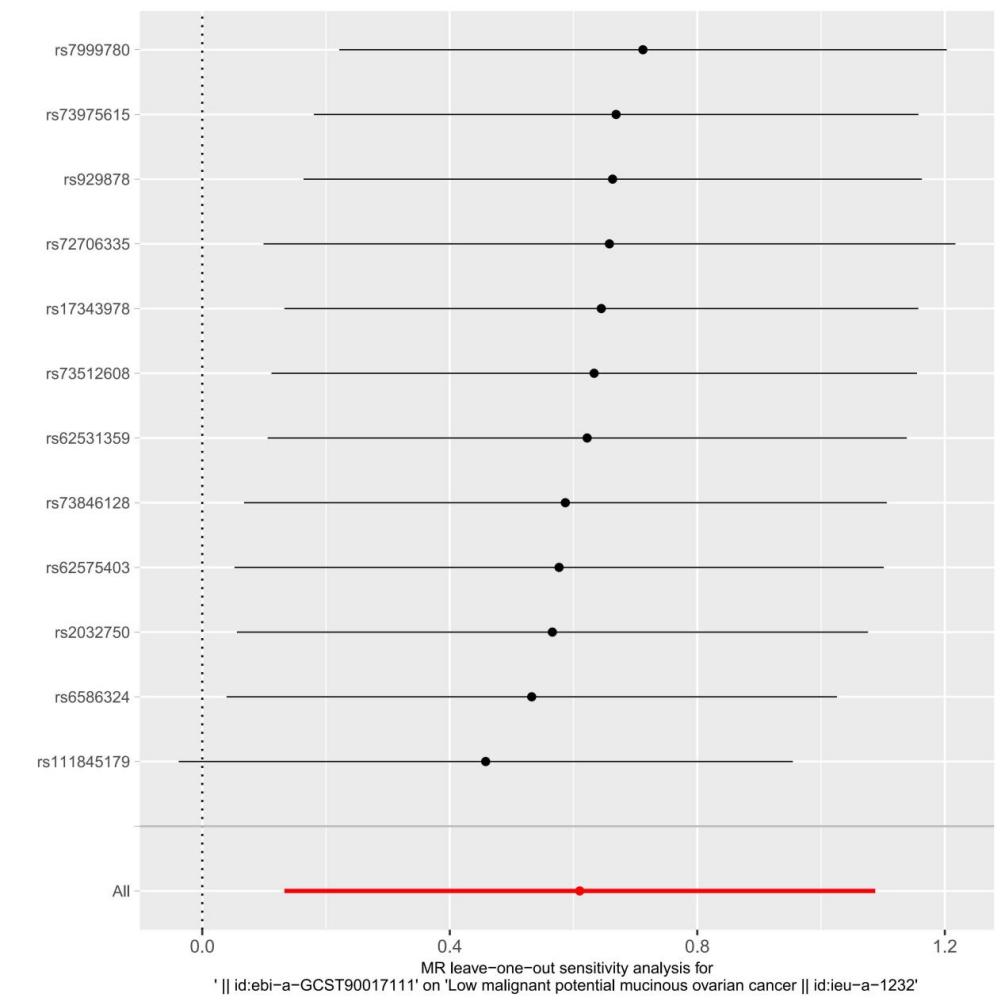

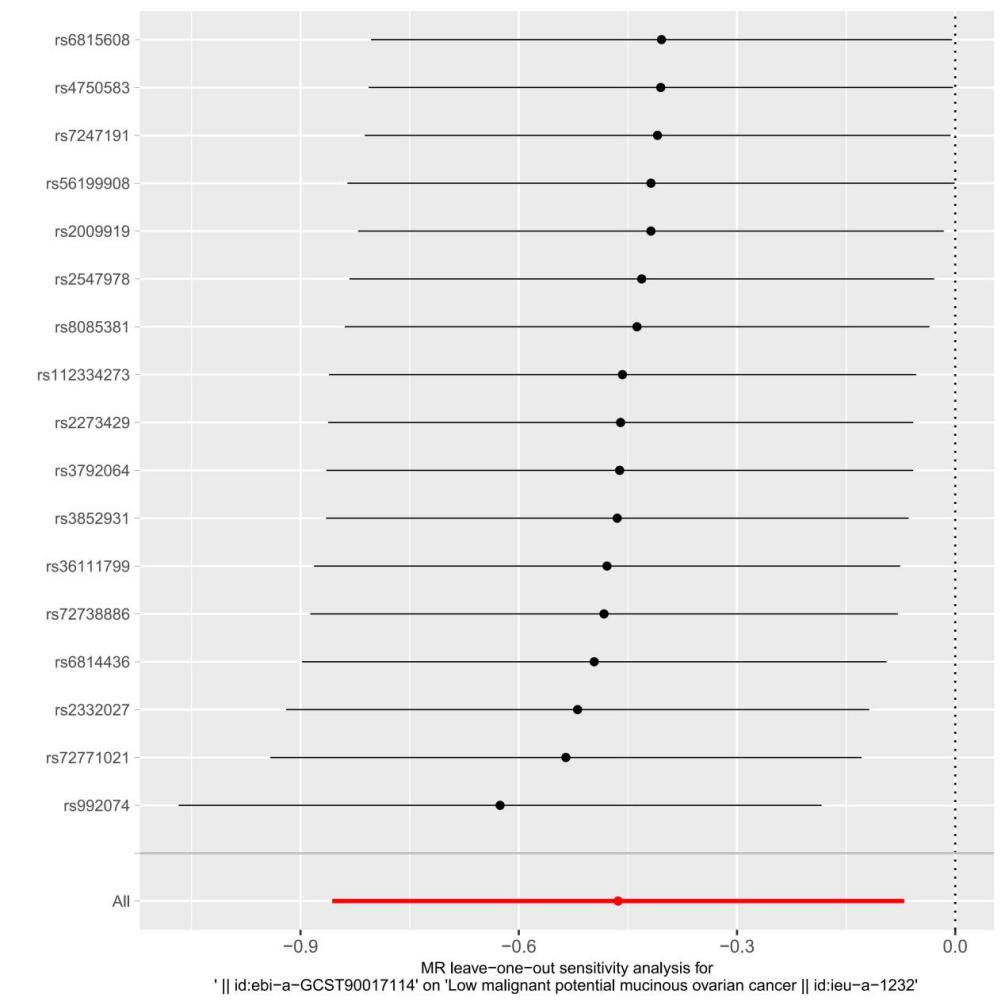

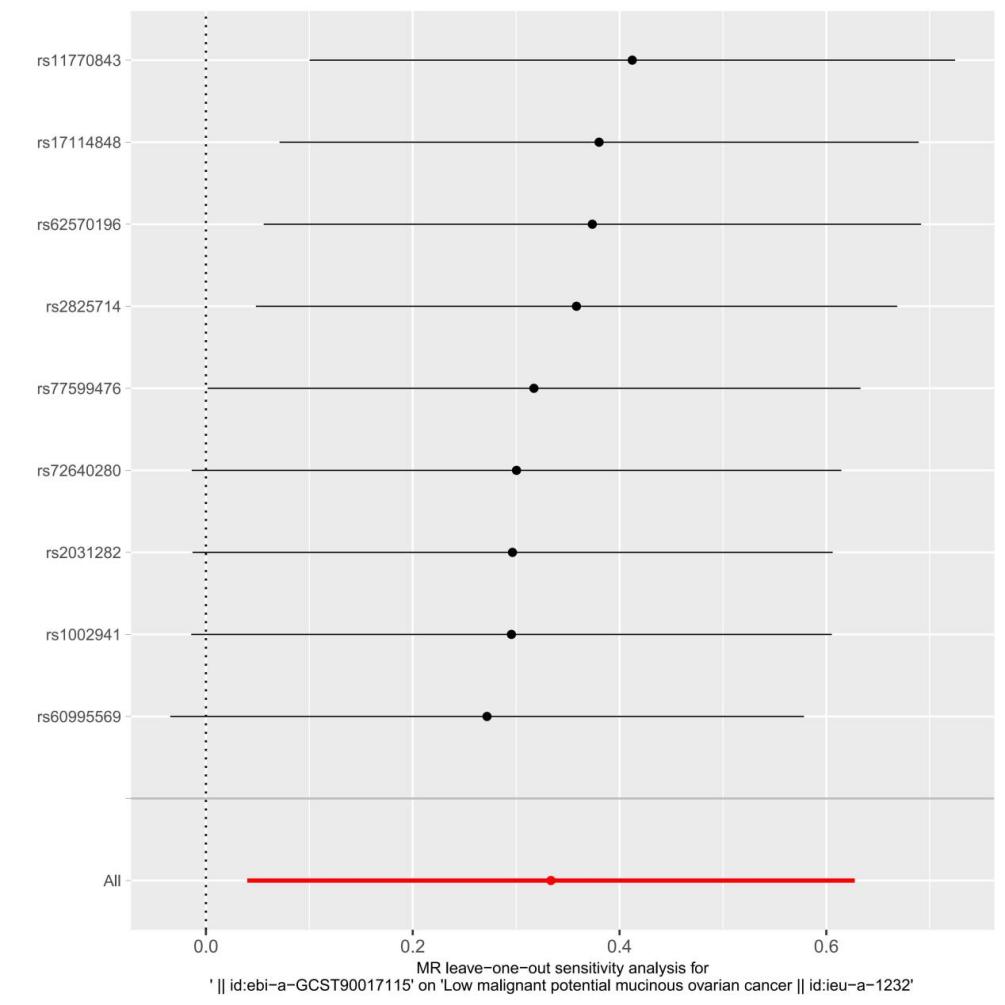

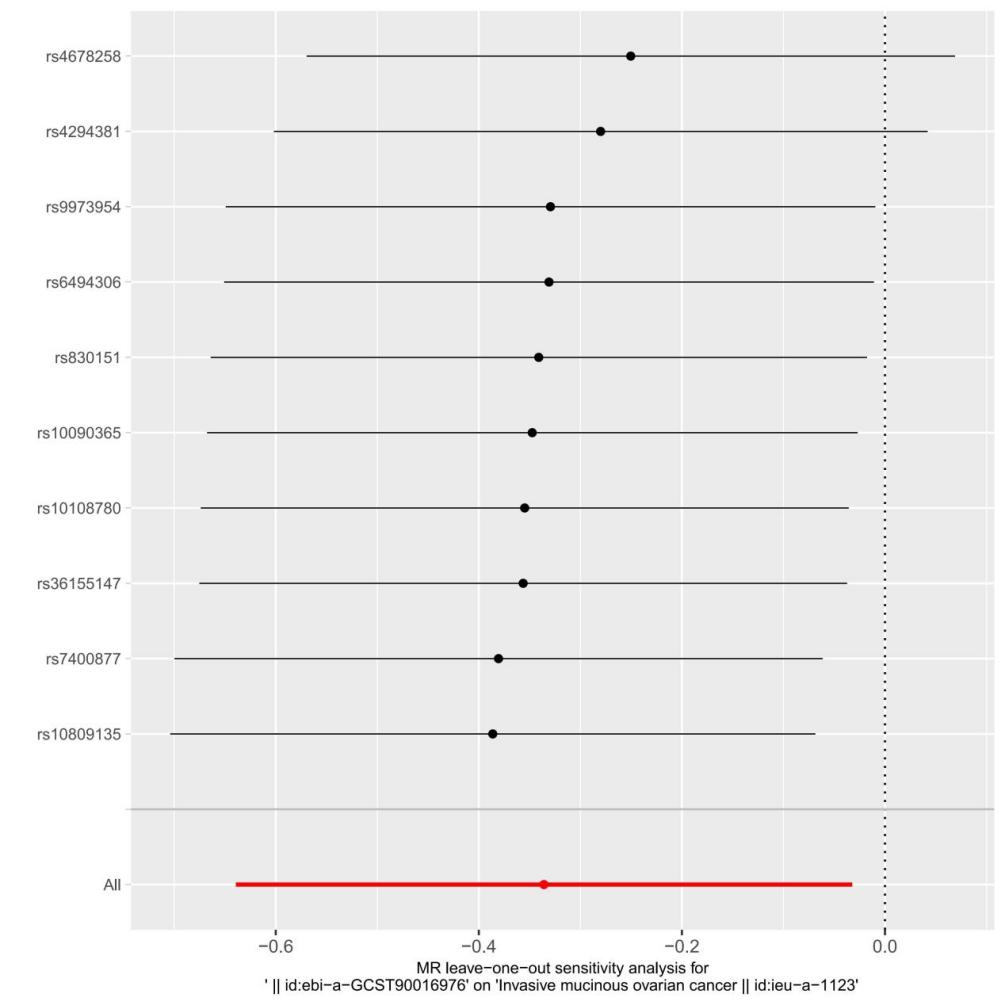

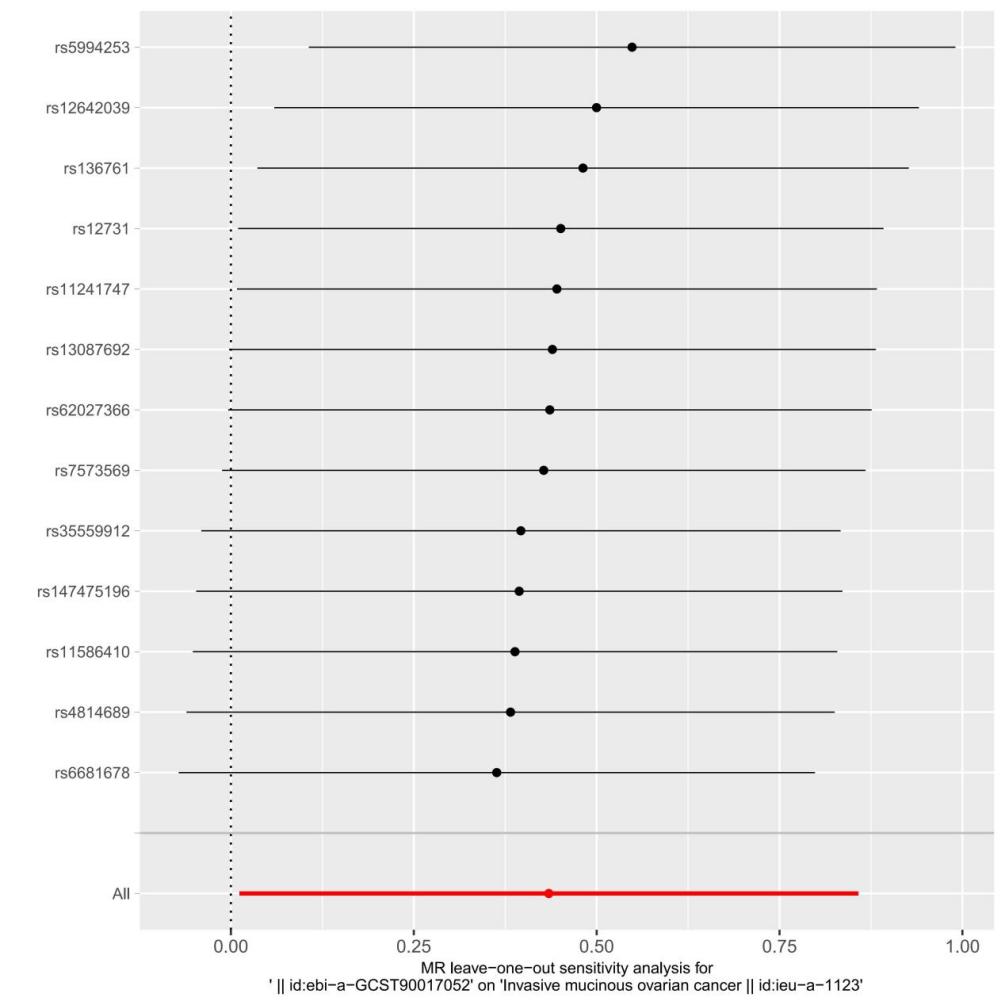

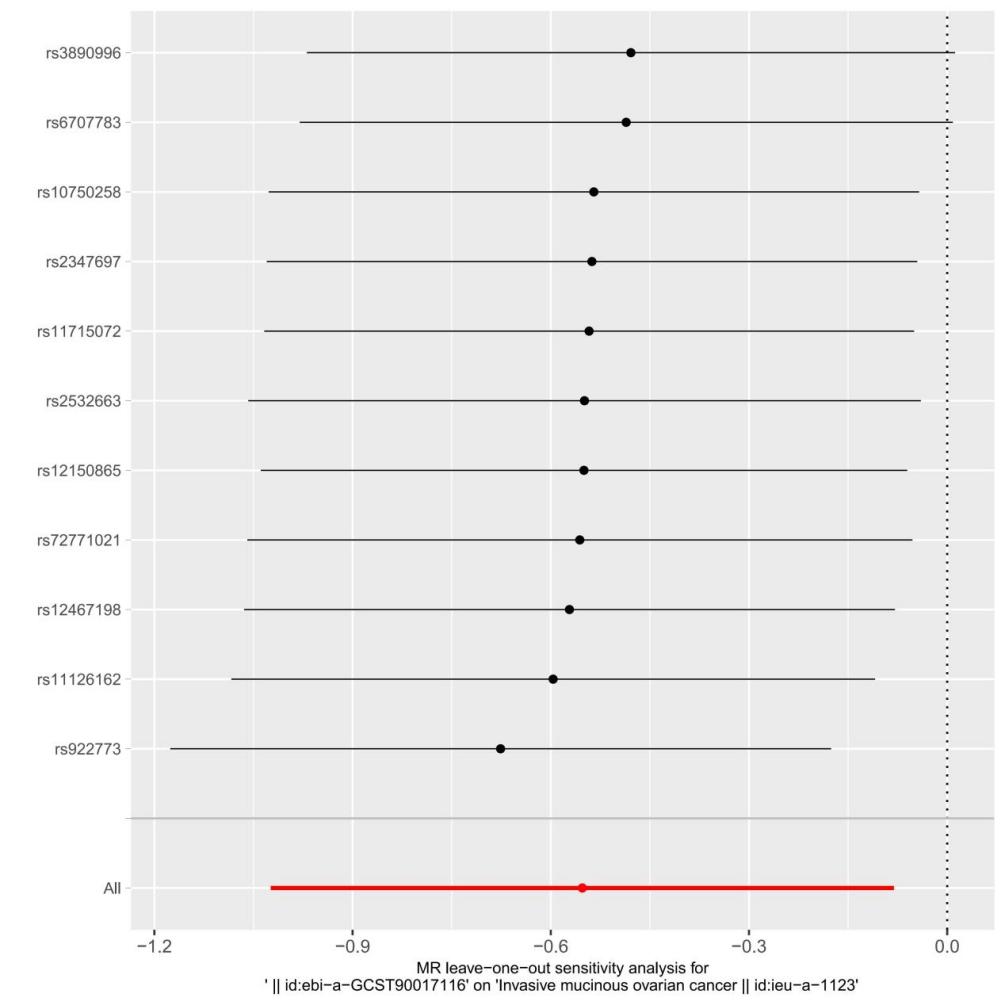


**2. Endometrial cancer**


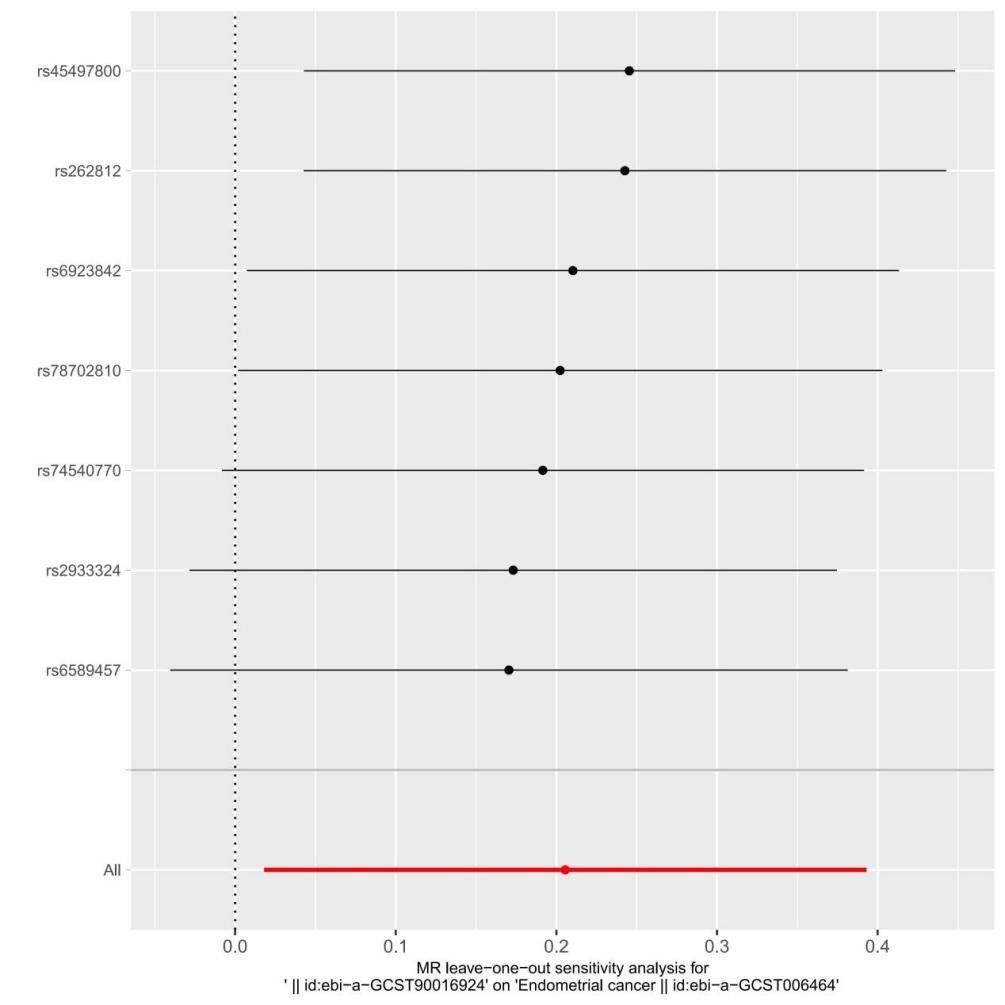

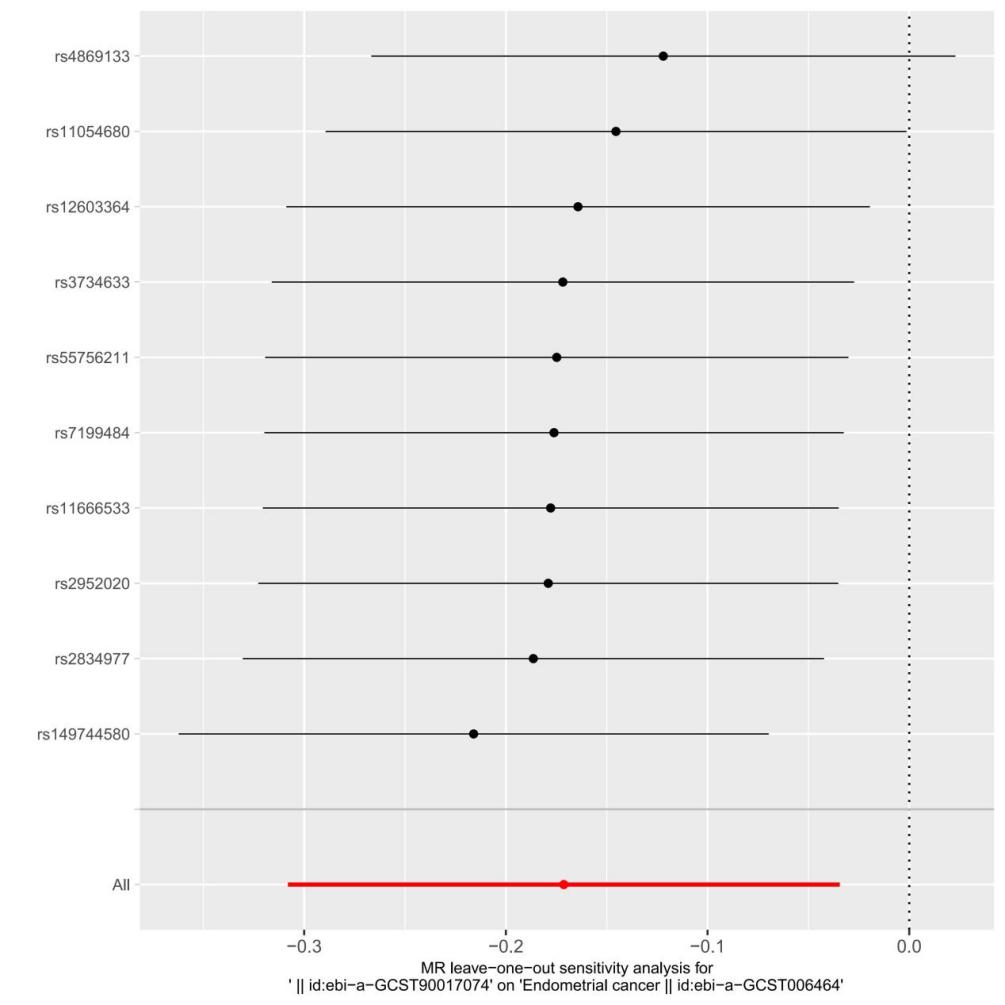

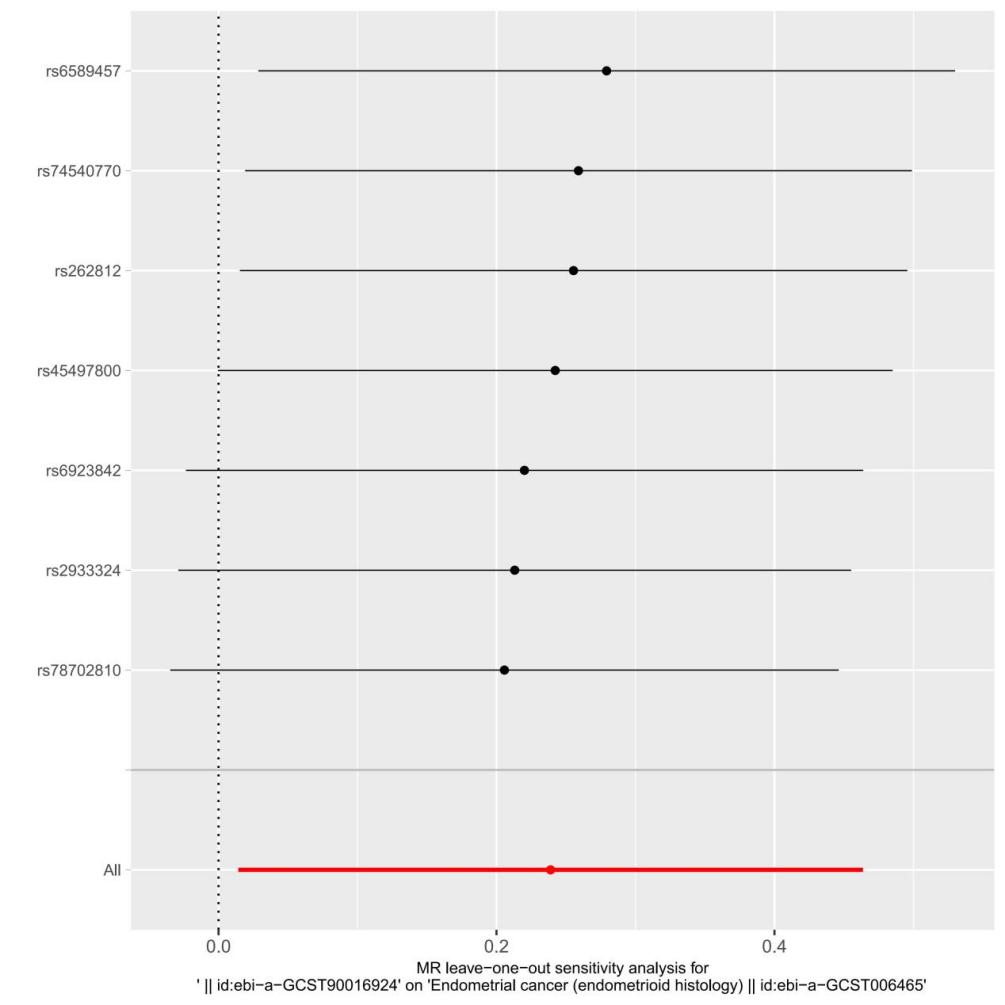

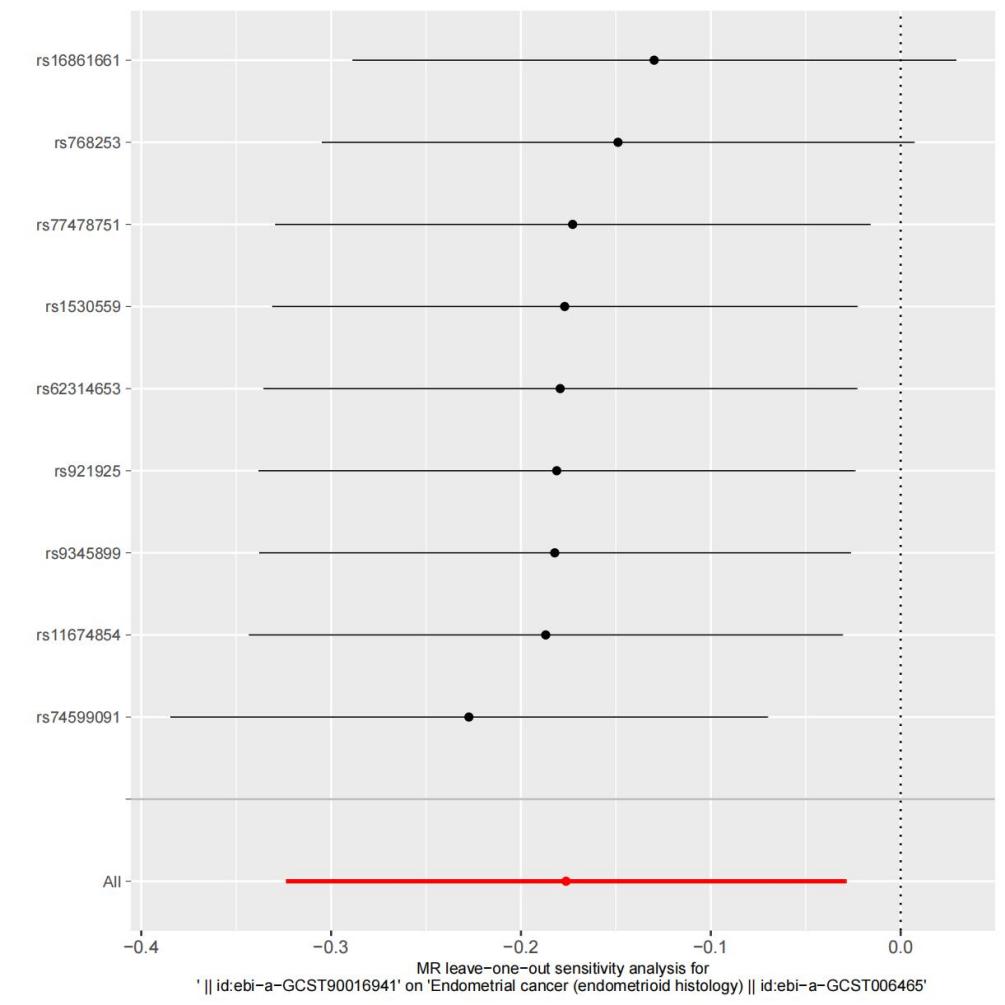

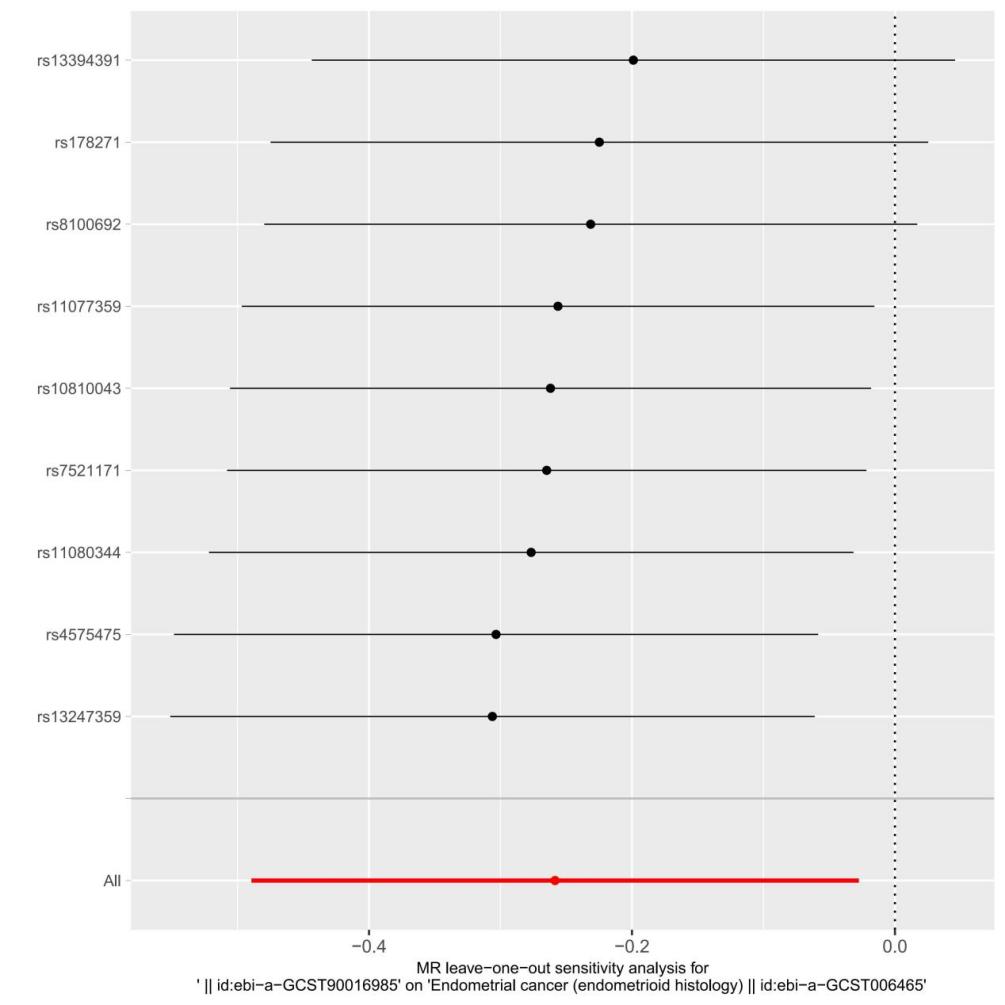

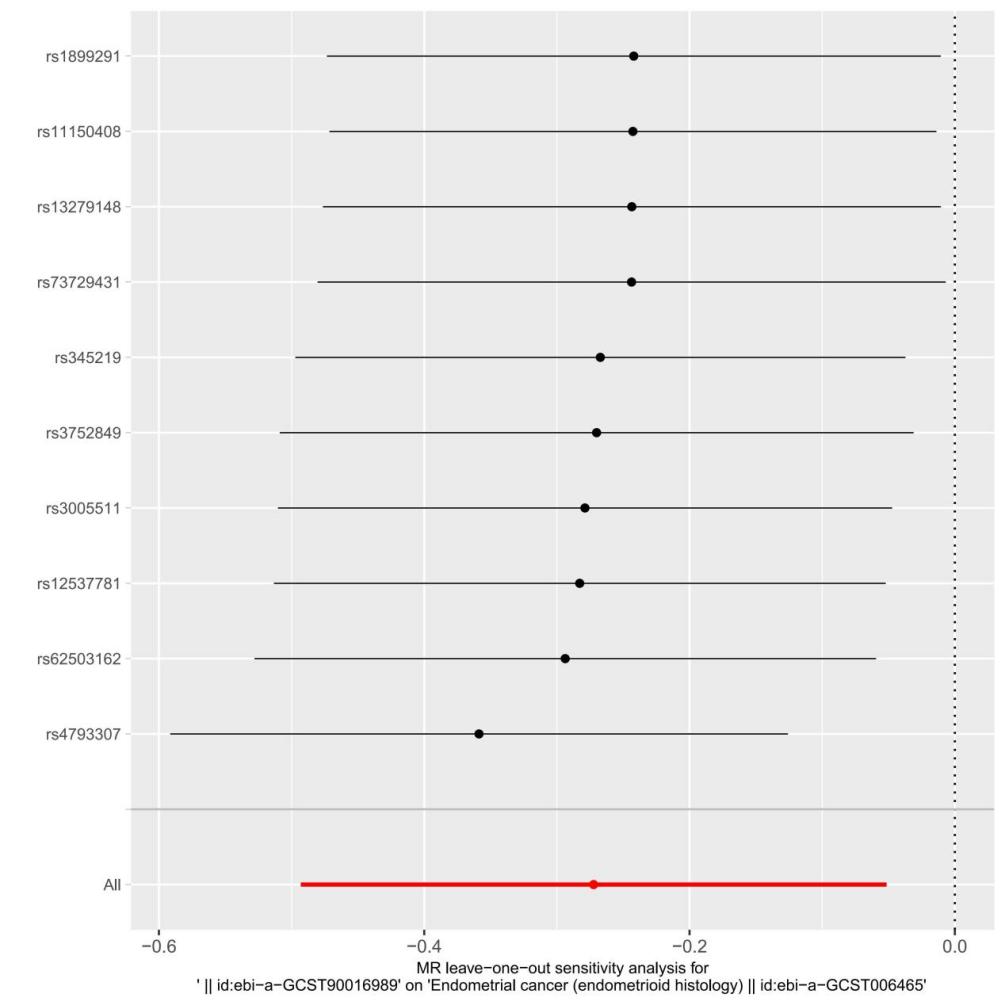

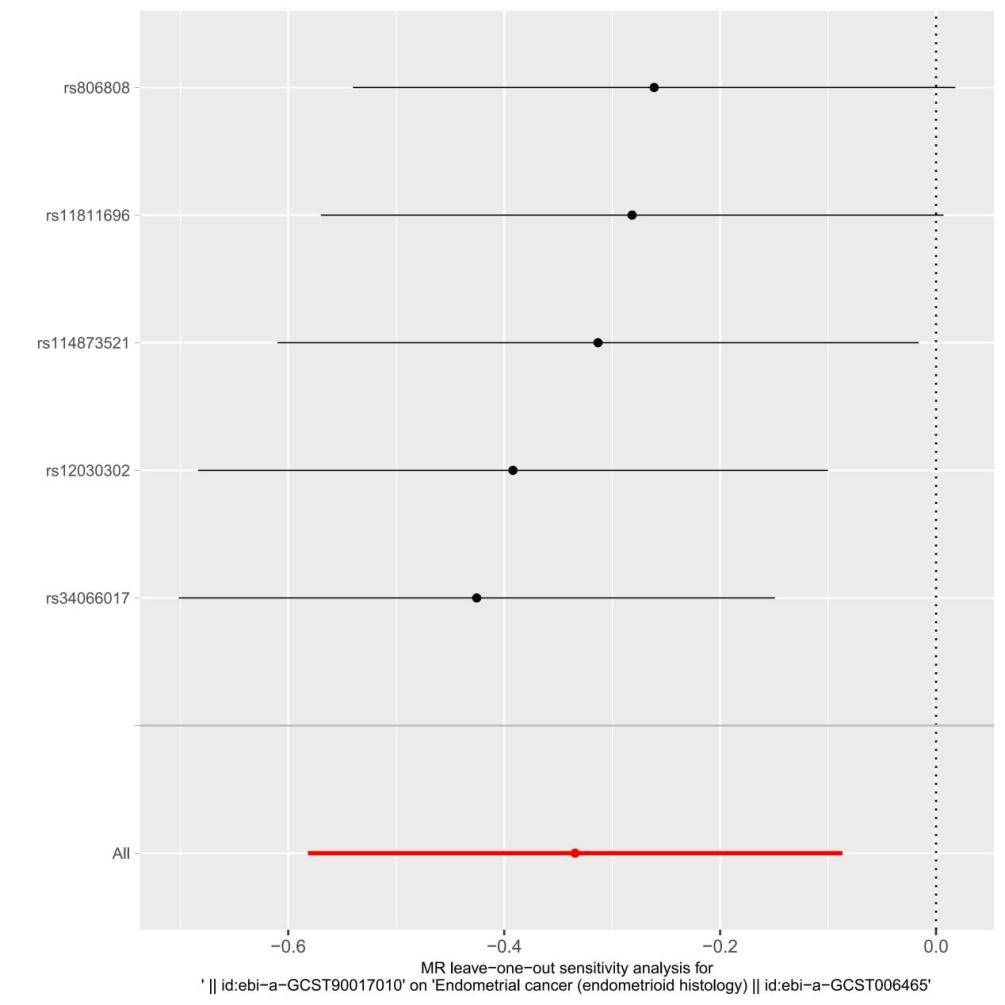

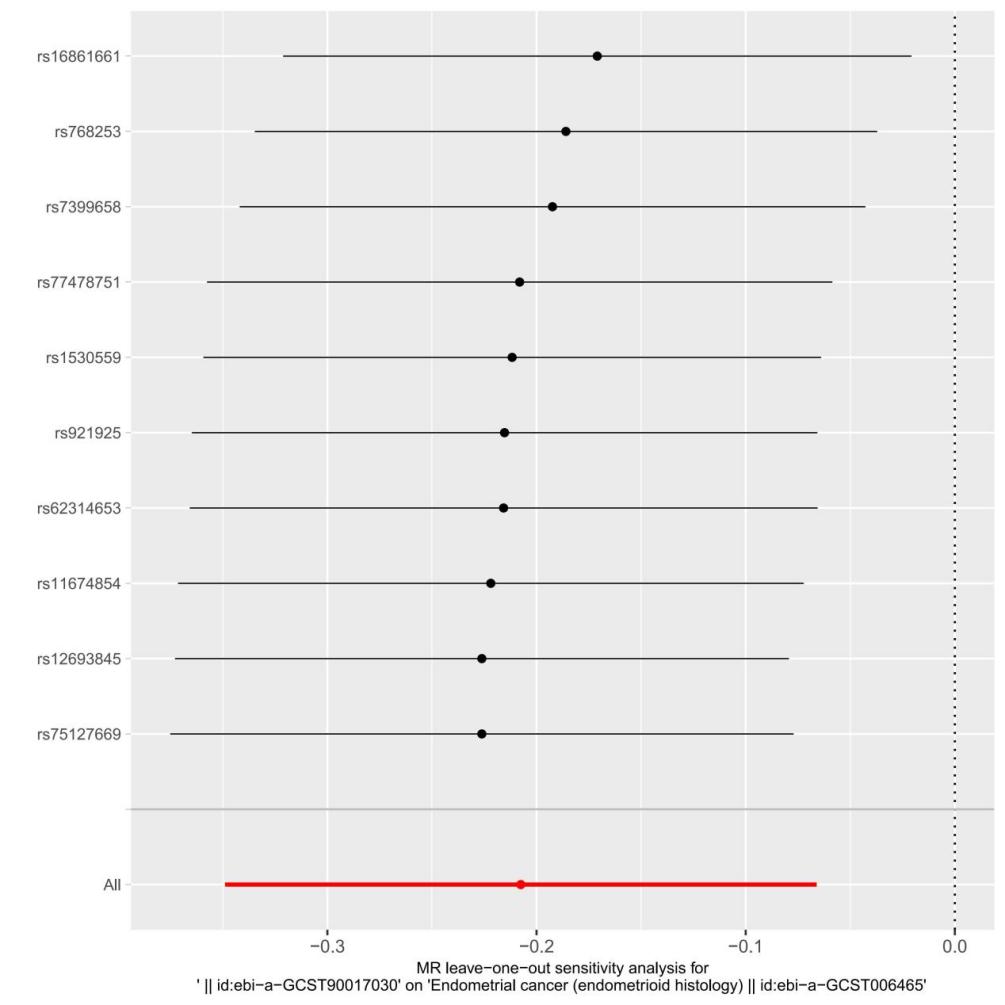

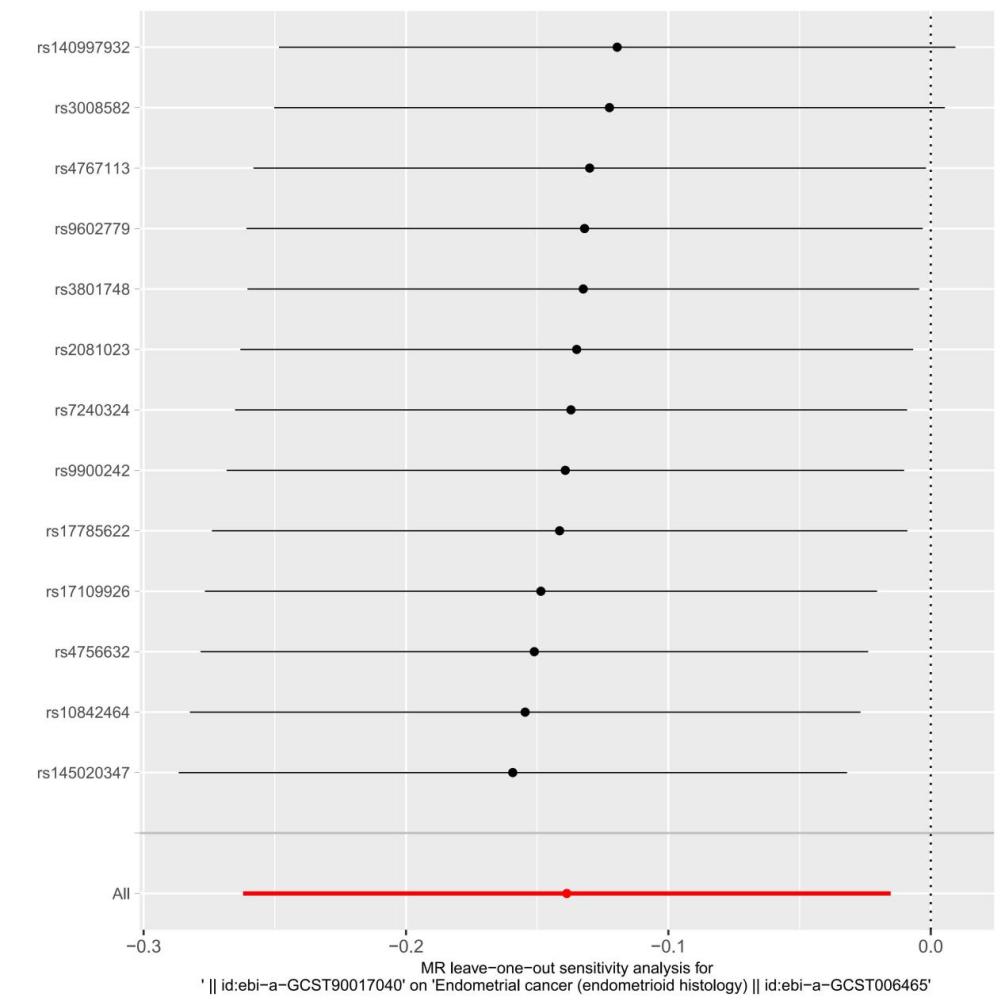

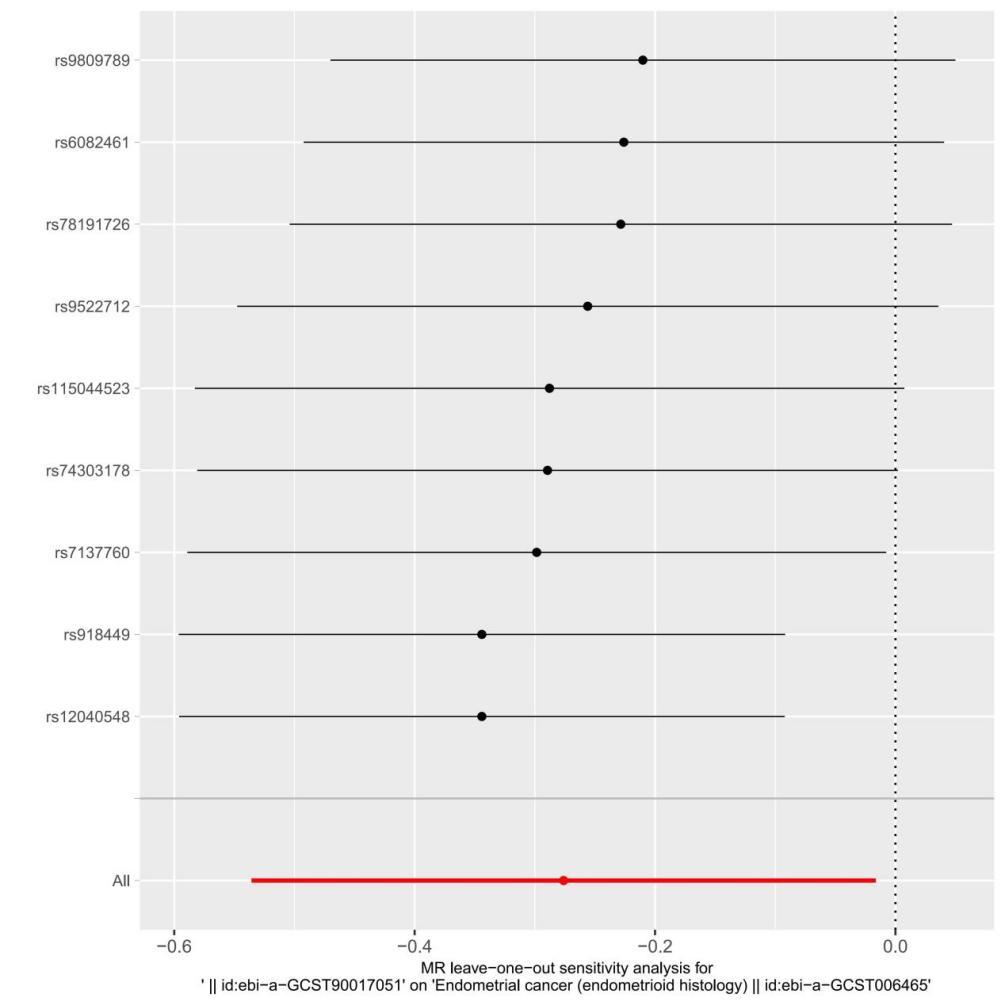

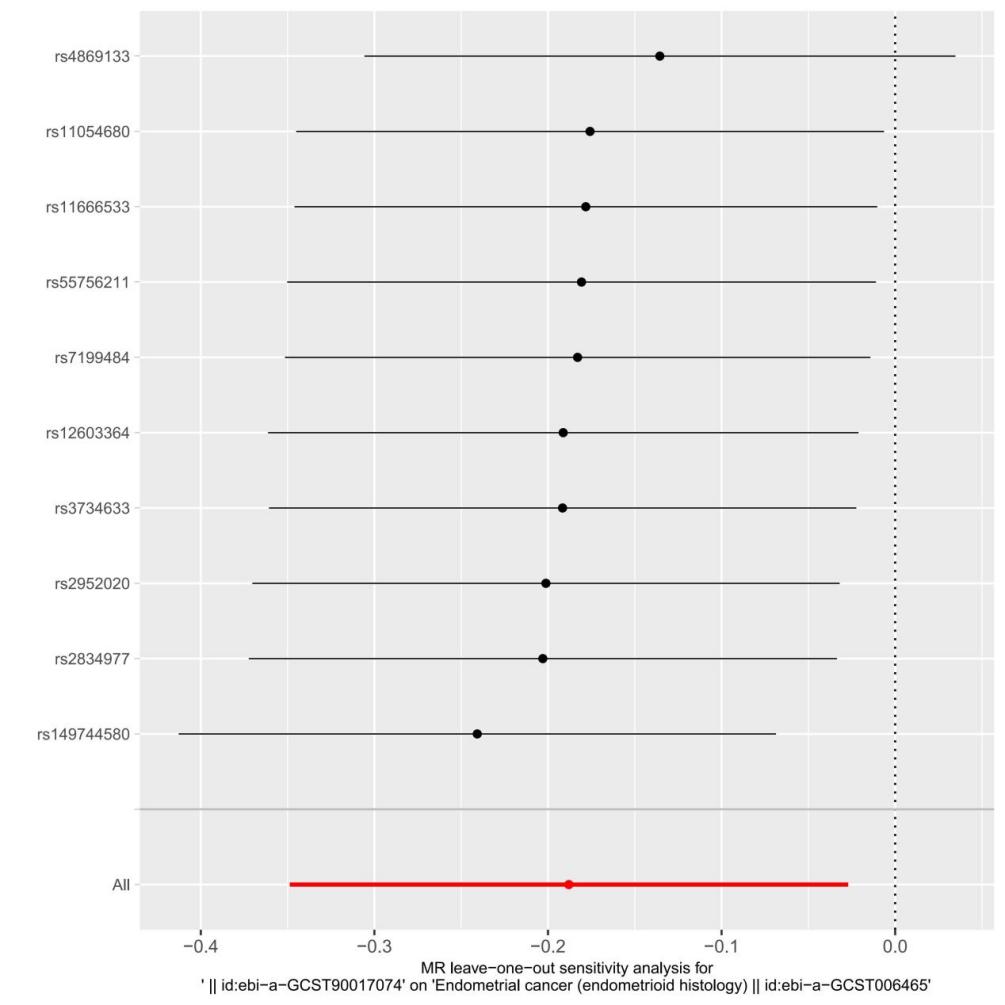

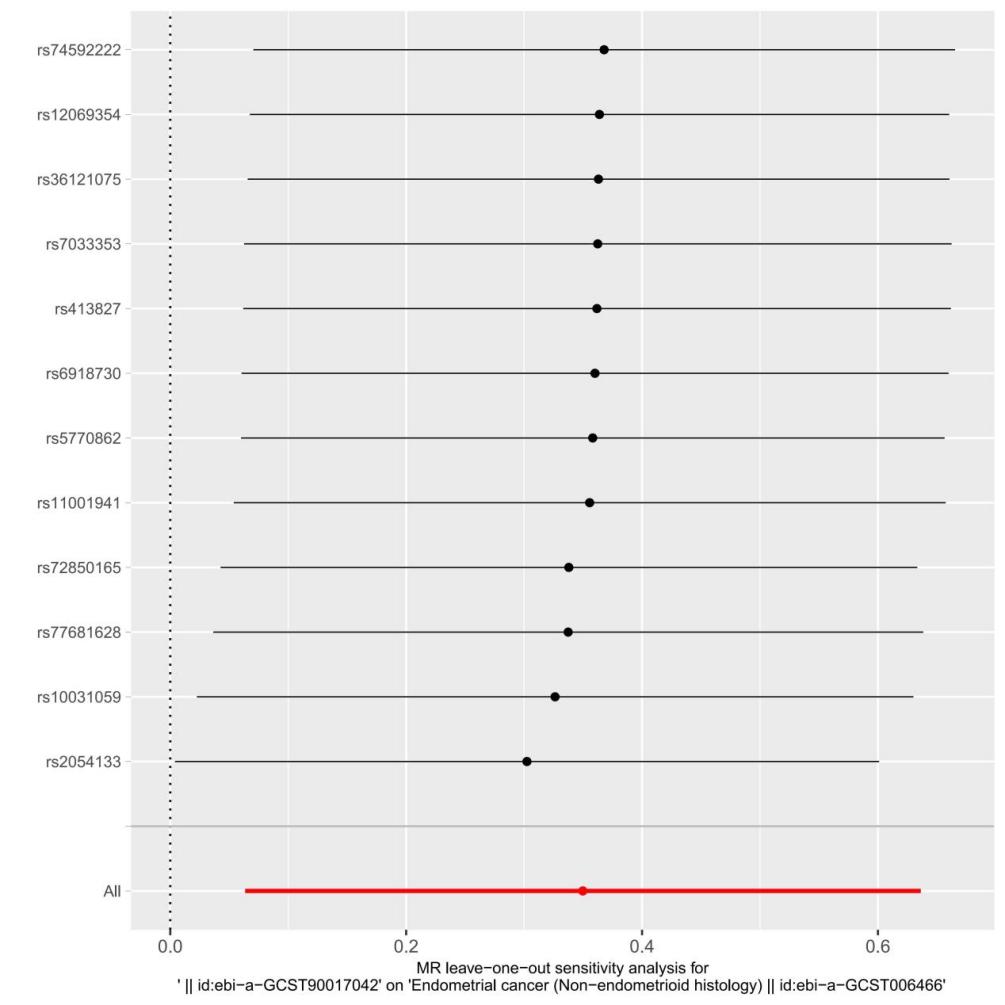


**3. Cervical cancer**


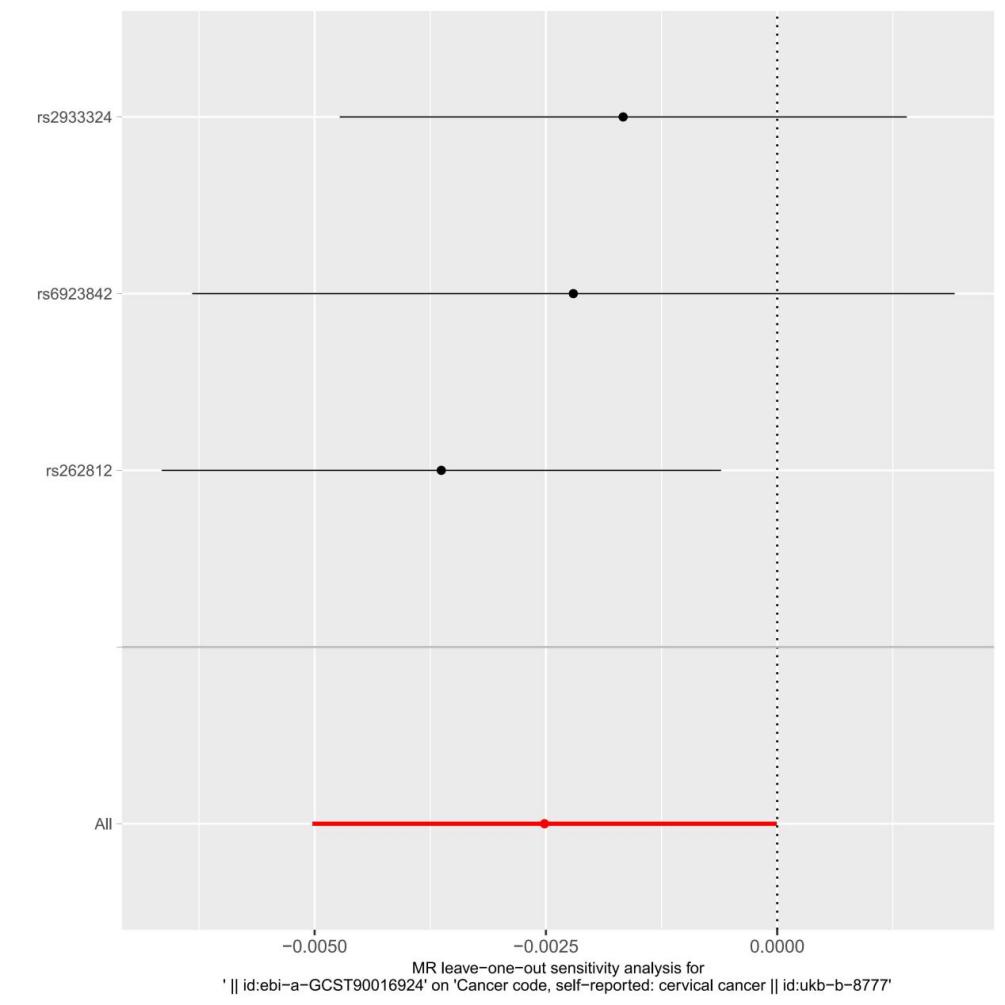

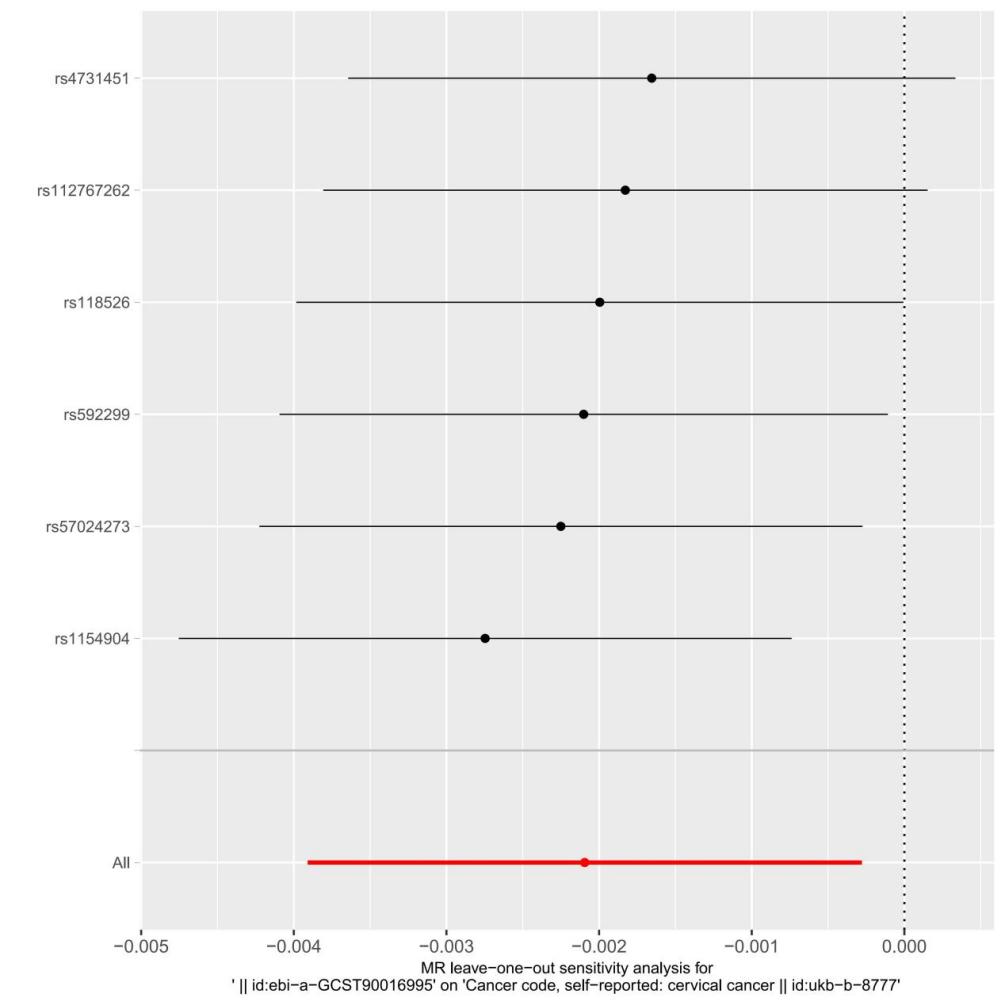

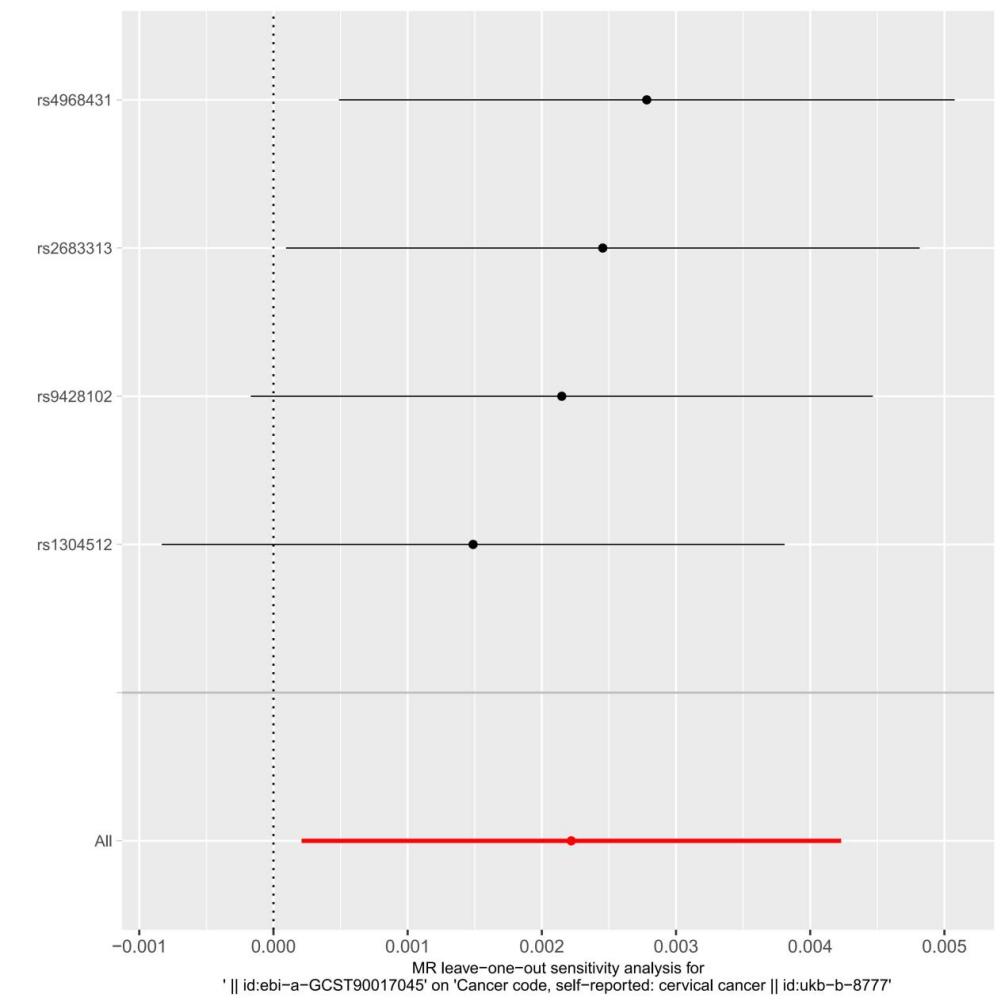

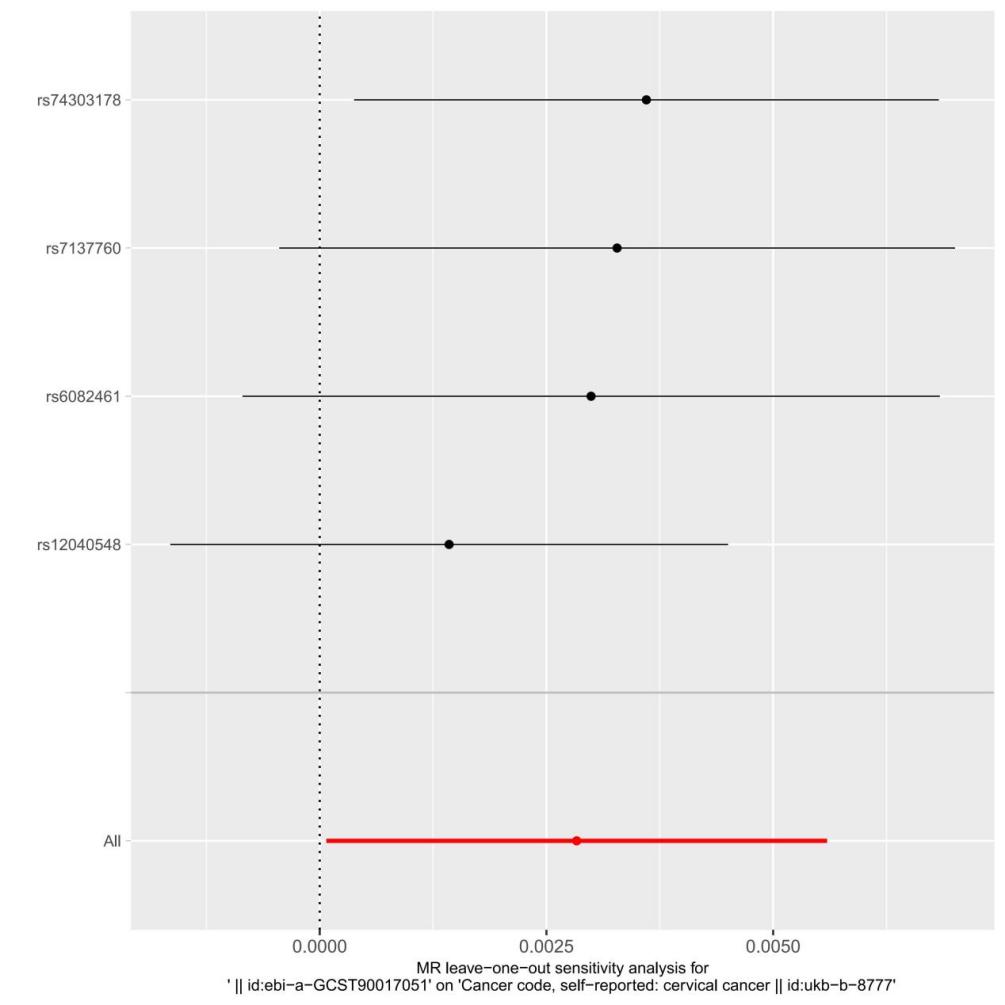

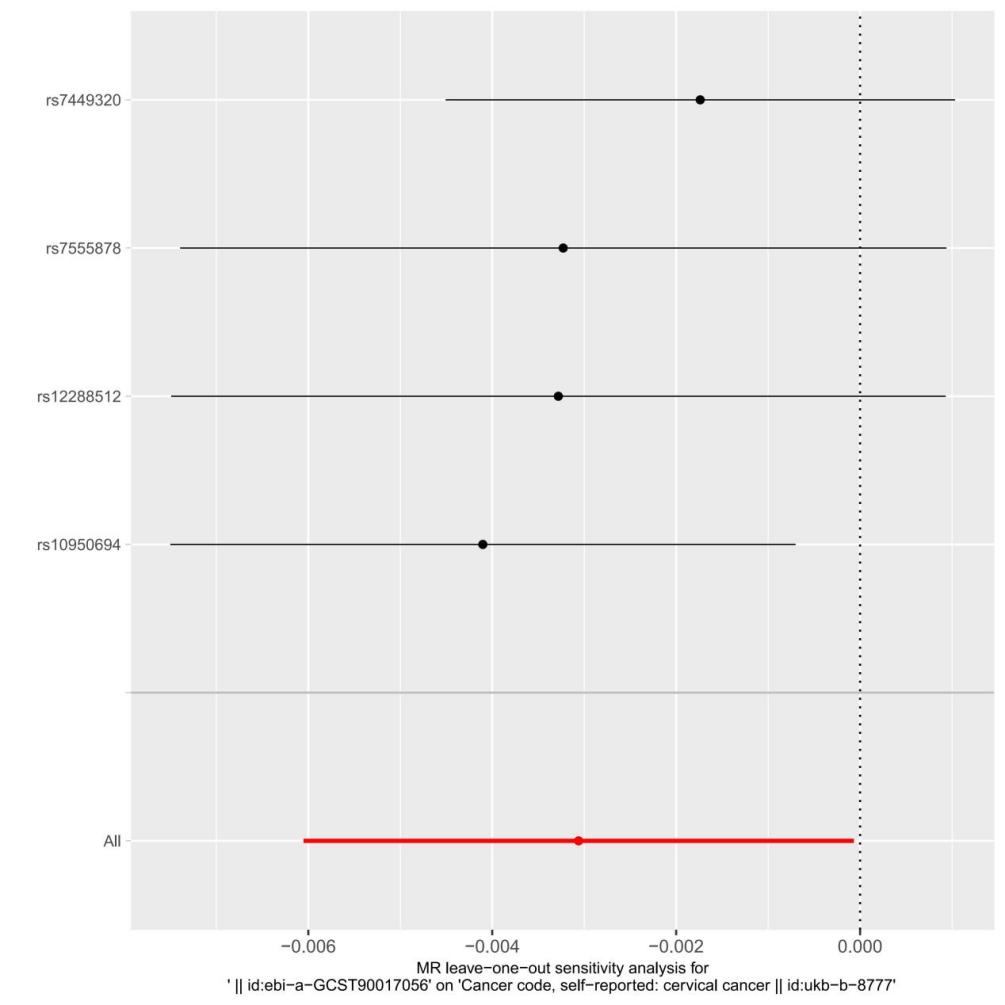

Supplement: Supplementary file 4 [file medi-103-e37628-s004.docx]
